# Chemical Proteomics Identifies Ketogenesis‐Mediated Cysteine Modifications Regulating Redox Function

**DOI:** 10.1002/anie.202519830

**Published:** 2026-01-16

**Authors:** Yuan‐Fei Zhou, Ling Zhang, Zhuoyi L. Niu, Xin Wang, Alejandro Storper, Ryan Hunt, Yingming Zhao, Nima Sharifi, Zhipeng A. Wang

**Affiliations:** ^1^ Desai Sethi Urology Institute & Sylvester Comprehensive Cancer Center University of Miami Miller School of Medicine Miami Florida 33136 USA; ^2^ Department of Microbiology and Cell Science Institute of Food and Agricultural Sciences University of Florida Gainesville Florida 32611 USA; ^3^ Ben May Department for Cancer Research The University of Chicago Chicago Illinois 60637 USA

**Keywords:** Cysteine modifications, Ketone body, Protein modifications, Proteomics, Redox regulation

## Abstract

All the studies of ketogenesis‐dependent post‐translational modifications (PTMs), notably those mediated by ketone bodies, β‐hydroxybutyrate (Bhb) and acetoacetate (Acac), have focused on lysine acylations. However, given the chemically diverse and reactive nature of metabolites generated, it remains unclear whether non‐lysine modifications can also happen. Here, we develop an acetoacetate‐alkyne (Acac‐alkyne) chemical probe that enables efficient metabolic labeling, robust fluorescent visualization, and site‐specific identification of Acac‐modified proteins. By combining chemical proteomics with open‐search strategy, we showed that Acac induces previously uncharacterized cysteine modifications in mammalian cells. Notably, cysteine crotonation (Ccr) is validated by employing both probe‐based and standard peptide‐based co‐elution assays. Metabolic pathway tracing further identifies BDH1 and ECHS1 as key enzymes that generate Ccr formation. We further demonstrate that Ccr at PRDX3 C229 site impairs dimerization and redox activity, linking this newly discovered modification to the regulation of cellular reactive oxygen species. Together, these findings establish ketone metabolism as a novel source of cysteine modifications and provide an alternative mechanistic pathway to explain the profound biological effects of ketone bodies.

## Graphical abstract

Herein, we report ketone body induce cysteine modifications beyond lysine acylation, as revealed by integrating chemical proteomics with open‐search methods. Among the modifications, cysteine crotonation (Ccr) was chemically validated by endogenous peptide‐based co‐elution assays. Metabolic tracing identifies BDH1 and ECHS1 as key Ccr‐generating enzymes. Functional studies showed that Ccr at C229 of PRDX3 impairs dimerization and redox function.

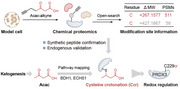

## Introduction

Ketogenesis, triggered by fasting, exercise, ketogenic diet or diabetic ketoacidosis, increases circulating ketone bodies β‐hydroxybutyrate (Bhb) and acetoacetate (Acac) to millimolar levels.^[^ ^1^, ^2^, ^3^, ^4^ ^]^ Ketone bodies are derived from acetyl‐CoA mainly in the liver and serve as metabolically efficient alternative energy sources, especially under conditions of glucose deprivation.^[^ ^5^ ^]^ After circulation, Bhb and Acac are taken up by extrahepatic tissues and converted into acetyl‐CoA, which subsequently enters the tricarboxylic acid cycle to produce ATP in tissues.^[^ ^6^ ^]^ In addition to serving as metabolic fuels, these ketones are converted to CoA derivatives that can be installed on histones and non‐histone proteins, leading to two recently identified marks: lysine β‐hydroxybutyrylation (Kbhb) and lysine acetoacetylation (Kacac).^[^ ^7^, ^8^ ^]^ These discoveries demonstrate that ketone bodies directly reshape the epigenetic landscape, bridging systemic metabolic status with gene regulation.^[^ ^9^, ^10^, ^11^ ^]^ Despite these advances, the effects of ketone‐driven PTMs beyond lysine acylations remain largely unexplored, leading to an incomplete understanding of ketogenesis at the molecular level.

To address this gap, we combine the chemical proteomics with an open‐search strategy to investigate if ketone bodies can be precursors for protein modifications other than lysine acylation. To this end, we synthesized a bioorthogonal probe of Acac (Acac‐alkyne) that closely mimics the chemical structure and biochemical reactivity of Acac. We then performed chemical proteomic profiling of Acac‐ modified proteins in living cells in a site‐specific manner, unveiling previously unknown cysteine (Cys) modifications induced by ketone bodies via open‐search. In order to confirm the modification structure of Cys + 267 (Cysteine plus 267.1577 Da), we used a probe‐based co‐elution assay to confirm that Cys + 267 is a novel cysteine crotonation (Ccr) modification. To demonstrate that this modification exists endogenously, we further confirmed this modification by parallel reaction monitoring (PRM) scanning and co‐elution experiments with synthetic peptides. We next sought to determine how Ccr is formed endogenously within cells. We proposed that Ccr is generated though Bhb and crotonyl intermediate. A probe‐based co‐elution assay confirmed that Bhb and crotonate (Cro) will also form the same Ccr modifications. Knockout of two key enzymes, BDH1 and ECHS1, resulted in decreased in‐gel fluorescence signals, indicating Ccr undergoes a reverse β‐oxidation like pathway to form Ccr.

To explore the biological consequence of Ccr, we focused on peroxiredoxin 3 (PRDX3), a thiol‐specific peroxidase, reducing hydrogen peroxide via disulfide bond formation between conserved cysteines.^[^ ^12^ ^]^ Our proteomic analysis revealed Ccr at the resolving cysteine C229, suggesting a potential disruption of PRDX3 activity. Functional assays demonstrated that Ccr increases PRDX3 thermal stability and impairs dimer formation. Acac treatment elevated cellular reactive oxygen species (ROS) levels, which were rescued only by overexpressing wild‐type (WT) PRDX3, but not by cysteine mutants (C229A or C229S) or the C229E, a mimic of C229cr, underscoring PRDX3 as a key redox target of this novel modification. Collectively, our findings reveal ketone bodies as a previously unrecognized driver of new cysteine modifications and provide an alternative mechanistic pathway to explain the profound biological effects of ketone body.

## Results and Discussion

### Design and Validation of an Acac‐Alkyne Probe for Metabolic Labeling

**Figure 1:**
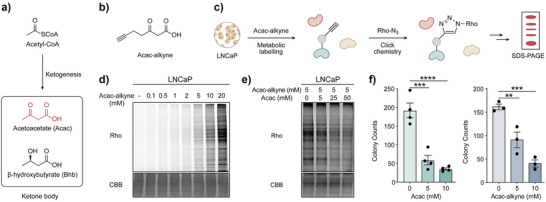
Design and validation of Acac‐alkyne probe for profiling Acac‐modified proteins. a) The ketone body produced from acetyl‐CoA via ketogenesis metabolism. b) Design of the bioorthogonal Acac derivative probe, Acac‐alkyne. c) In‐gel fluorescence strategy for detection of Acac‐modified proteins using Acac‐alkyne. d) LNCaP cells were labeled with increasing concentrations of Acac‐alkyne, showing a dose‐dependent manner. Coomassie Brilliant Blue (CBB) staining was used as a loading control. e) Co‐treatment with increasing concentrations of Acac attenuated the labeling signal of Acac‐alkyne. f) Acac and Acac‐alkyne exhibited similar colony‐suppressive effects. Acac (*n* = 4; mean ± SEM; ***p < 0.001, ****p < 0.0001, one‐way ANOVA); Acac‐alkyne (*n* = 3; mean ± SEM; **p < 0.01, ***p < 0.001, one‐way ANOVA). The CBB images in panels d and e are compressed versions of the corresponding gels used for Rho staining; the corresponding uncropped Rho‐stained and corresponding CBB images are shown in Figure S7 within the Supporting Information.

Bioorthogonal chemical probes provide a robust platform for interrogating PTMs.^[^ ^13^, ^14^ ^]^ Probes bearing alkyne or azide tags can be metabolically incorporated into the corresponding native PTM sites on endogenous proteins in cells.^[^ ^15^, ^16^, ^17^, ^18^ ^]^ A subsequent click reaction^[^ ^19^, ^20^ ^]^ conjugates fluorescent dyes or biotin to these tags, enabling selective visualization and identification of the labeled proteins. Ketogenesis, triggered by fasting, exercise, or a ketogenic diet, elevates circulating ketone body levels to the millimolar range through acetyl‐CoA metabolism (Figure 1a). Accordingly, we hypothesize that an alkynyl analogue of Acac will enter this metabolic pathway and incorporate into Acac‐modified proteins in live cells. We thus designed and synthesized an Acac derivative probe, Acac‐alkyne, containing a terminal alkynyl group (Figure 1b). Acac‐alkyne was synthesized from 4‐pentynoic acid (compound **1**) and Meldrum's acid to generate compound **2** (Figure S1a), which was converted to compound **3** through *tert*‐butanol mediated esterification, and subsequent deprotection to form the final Acac‐alkyne probe (compound **4**).

With Acac‐alkyne in hand, we first evaluated whether this probe can be metabolically incorporated into proteins in mammalian cells. To this end, LNCaP prostate cancer cells, used as a model cell line, were treated with Acac‐alkyne for 24 hours. After harvesting the cells, the whole cell lysates were subjected to azide‐alkyne click chemistry to conjugate the Acac‐alkyne labeled proteins to a rhodamine dye (Rho). The labeled proteins were then resolved by SDS‐PAGE gel and visualized by in‐gel fluorescence (Figure 1c). Our experiment demonstrated that a wide range of proteins was labeled by Acac‐alkyne in a dose‐dependent manner, with 5–10 mM as optimal concentrations (Figures 1d, S1b, S7, S8). Similar metabolic labeling results were observed across multiple cell types, including HEK293T, 22Rv1, Hepa1‐6, and NIH3T3 (Figures S1c, S8). Our result highlights the general applicability of Acac‐alkyne and excellent labeling efficiency. In addition, when cells were treated with a mixture of Acac and Acac‐alkyne for 2 hours, the fluorescence signal of the probe‐labeled proteins decreased with increasing concentration of Acac in a dose‐dependent manner. These results indicated that Acac‐alkyne effectively mimics Acac and was incorporated into Acac‐modified proteins (Figures 1e, S7). Functionally, Acac has been reported to significantly reduce colony formation in cancer cells.^[^ ^21^ ^]^ Consistently, we also observed that Acac significantly inhibited colony formation after treatment, and Acac‐alkyne recapitulated this effect (Figure 1f). In contrast, Bhb showed no such activity (Figure S1d, e), indicating that Acac‐alkyne mimics Acac, but not Bhb. Collectively, these findings validated the Acac‐alkyne as a bioorthogonal chemical probe to profile Acac‐modified proteins.

### Combining Chemical Proteomics and Open‐Search Strategy to Characterize Unknown Cysteine Modifications

**Figure 2:**
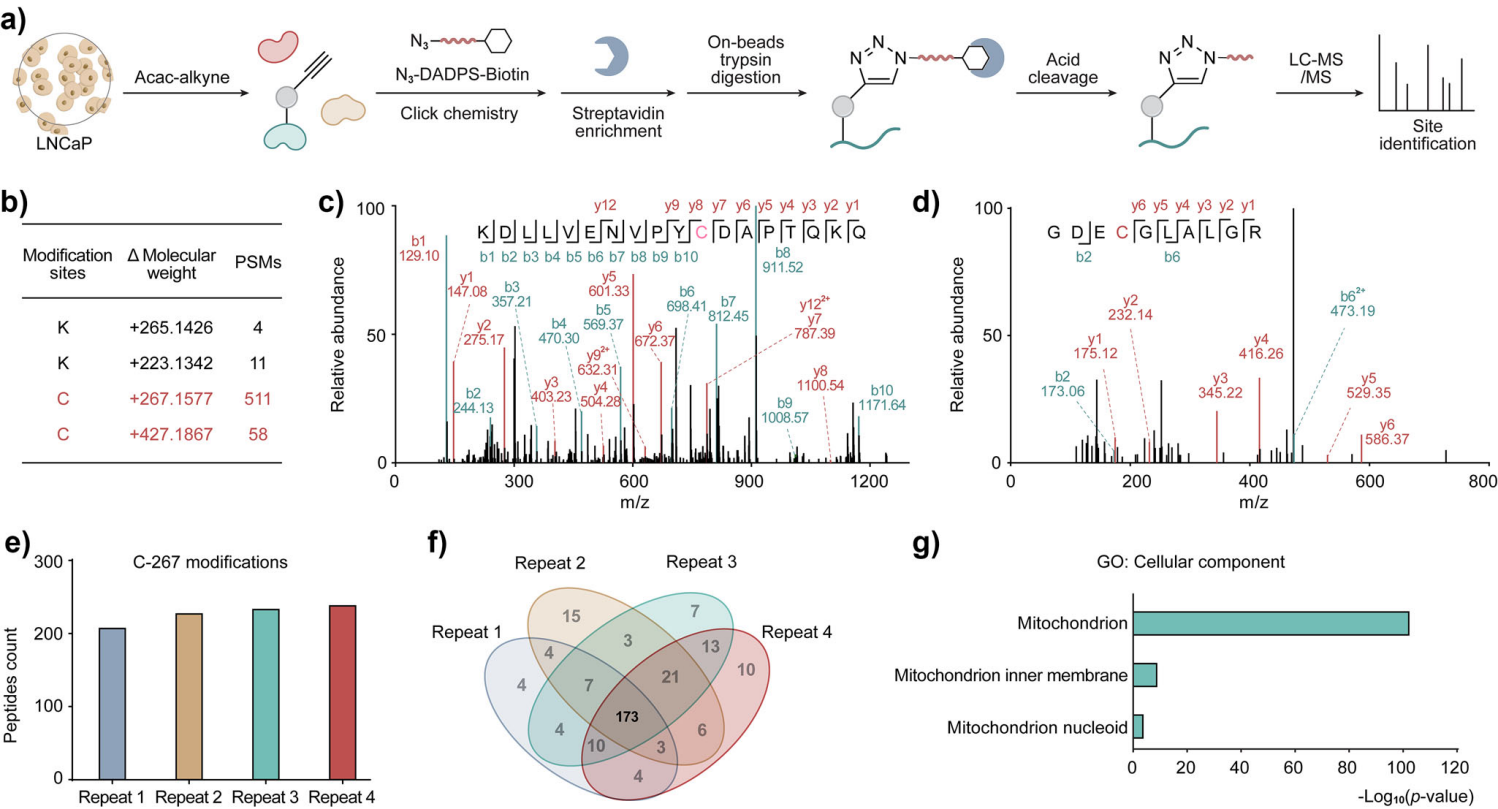
Combining chemical proteomics and an open‐search strategy identified novel cysteine modifications. a) TOP‐ABPP workflow for the site‐specific chemical profiling of Acac‐modified proteins using Acac‐alkyne. b) Open‐search strategy characterized two unknown cysteine modifications, each associated with distinct PSM counts. c, d) A representative MS2 spectrum shows the Cys + 267 (LYRM C96) and Cys + 427 (ALDH18A1 C247) modifications, respectively. e, f) The chemical proteomics data indicated 173 peptides containing Cys + 267 modification across four replicates. g) GO enrichment analysis of identified Cys + 267 modifications are exclusively represented in the mitochondrion.

After verifying the effectiveness of the Acac‐alkyne probe, we performed site‐specific chemical proteomics study to unveil Acac‐modified proteins at the proteome level by tandem orthogonal proteolysis‐activity‐based protein profiling (TOP‐ABPP) methods.^[^ ^22^, ^23^ ^]^ Lysates from Acac‐alkyne‐labelled LNCaP cells were prepared, conjugated with the acid‐cleavable azido‐DADPS‐biotin tag, followed by affinity purification, on‐bead digestion and acid‐mediated cleavage to enrich modified peptides (Figure 2a). The eluted Acac‐alkyne modified peptides were analyzed by LC‐MS/MS. We first performed an open‐search strategy^[^ ^24^, ^25^ ^]^ to identify any potential modifications that may occur. As anticipated, we have found lysine‐derived modification representing Kacac (K, + 265.1426 Da) (Figure 2b). Notably, we also found two previously unknown cysteine modifications with adduct masses are cysteine + 267.1577 Da (Cys + 267) and + 427.1867 Da (Cys + 427) (Figure 2b). These results drew our attention, as Acac has not previously been reported to induce any cysteine modifications. We manually verified the representative MS2 spectra for the modified peptides, such as Cys + 267 modification at LYRM C96 (KDLLVENVPYCDAPTQKQ) and Cys + 427 modification at ALDH18A1 C247 (GDECGLALGR), confirmed high confidence in identification (Figure 2c,d). To improve reliability and coverage of the modified cysteine identification, we subsequently conducted a closed search with only the sites identified in all four biological replicates considered for further analysis. The refinement resulted in 173 peptides containing Cys + 267 modification (Figure 2e,f), corresponding to 120 distinct proteins (Figure S2a). Cellular component analysis using the Gene Ontology (GO) database revealed that Cys + 267 modifications are exclusively localized in the mitochondria (Figure 2g), with associated biological processes involving tRNA processing and protein biosynthesis (Figure S2b). Similarly, 35 peptides containing Cys + 427 modification, also mapping to 29 proteins were detected in all four biological repeats (Figure S2c,d). Cellular component analysis again revealed that Cys + 427 modifications are exclusively localized in the mitochondria (Figure S2e). Molecular function analysis showed that the modified proteins are enriched in ribosomal protein, ribonucleoprotein, and oxidoreductase (Figure S2e). Next, we treated LNCaP cells with 10 mM Acac or Acac‐alkyne for 24 hours and performed dimethyl‐labeling‐based quantitative proteomics (Figure S2f). We assigned proteins with enrichment values (Log_2_L/H, light/heavy ratio) > 1 and identified 398 enriched proteins. This dataset includes 33 proteins sharing the same proteins identified in our site‐specific chemoproteomic analysis (Figure S2a), including ACAT1, HSD17B10, PRDX3, ACADVL, FMC1, and others (Figure S2g,h). Collectively, combining chemical proteomics and open‐search strategy, we characterized two novel cysteine modifications that exclusively distributed in mitochondria, significantly expanding the chemical space of ketone body‐derived PTMs and its potential role in metabolic regulation.

### Confirmation of the Structure of Cys + 267 Modification by Probe‐Based Co‐Elution Assay

**Figure 3:**
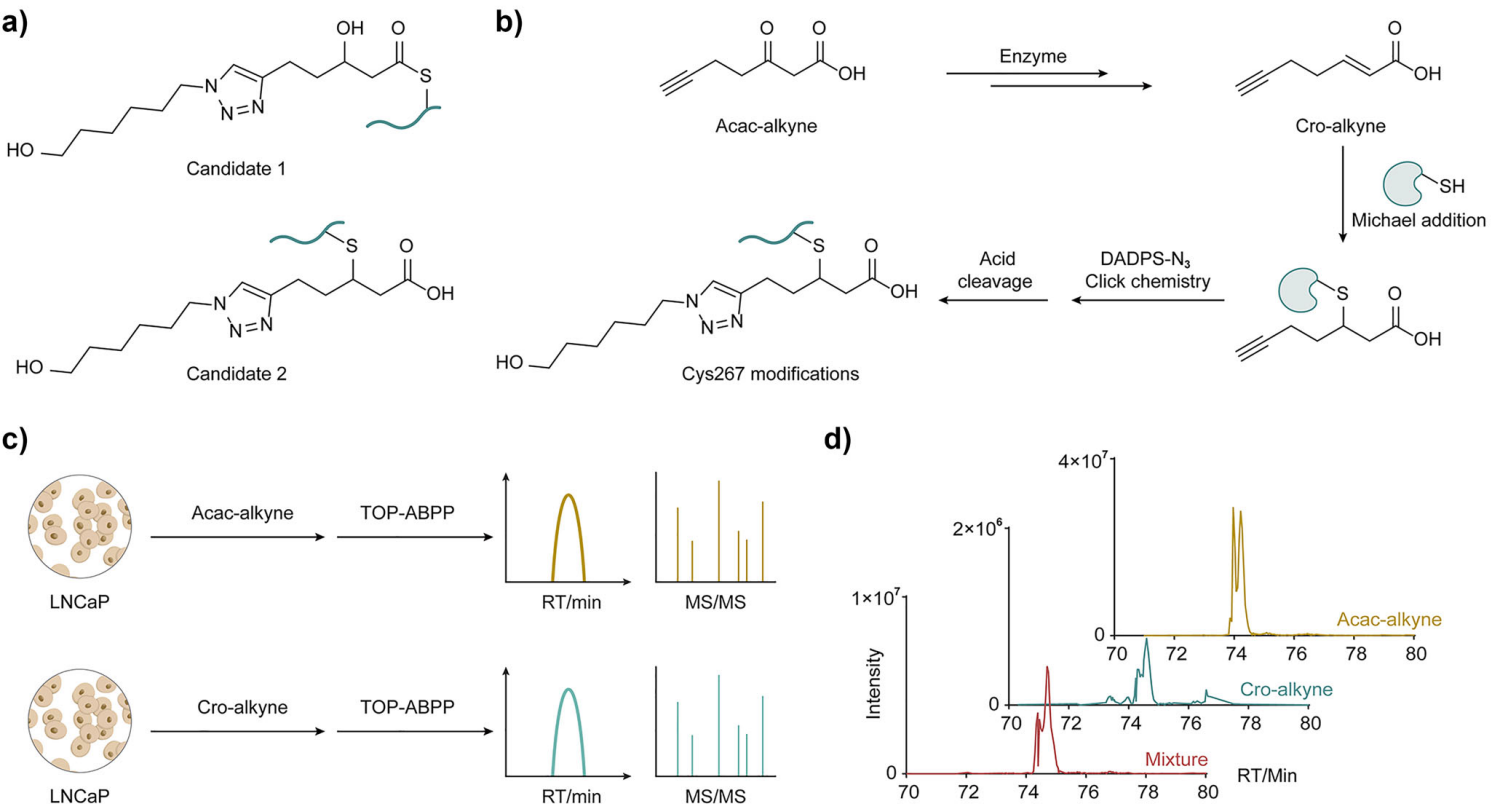
Confirmation of the structure of Cys + 267 modification by probe‐based co‐elution assay. a) Structures of two potential candidates bearing Cys + 267 modification. b) Acac‐alkyne is proposed to be transformed to Cro‐alkyne, followed by Michael addition with cysteine residues to form Cys + 267 modification. c) Comparing retention time and MS characteristics of Cro‐alkyne and Acac‐alkyne‐modified peptides by TOP‐ABPP assay. d) Retention time of the extracted ion chromatogram corresponding to the Cys + 267 modification of PRDX3 C229 in Acac‐alkyne, Cro‐alkyne and mixture groups.

Given that Cys + 267 exhibited the highest number of modification sites, we prioritized its structural characterization. Based on the observed Cys + 267 mass shift, there are two potential candidates (Figure 3a) formed via different metabolic pathways. For candidate 1, Acac‐alkyne was hypothesized to undergo carbonyl reduction followed by thioester formation with protein cysteine residues to generate Cys + 267 modification (Figure S3a). However, thioesters are generally unstable under basic conditions and prone to hydrolysis. To assess this possibility, we treated Acac‐alkyne labeled samples with or without 200 mM hydrazine^[^ ^26^ ^]^ (Figures S3b, S9), and observed no change in fluorescence intensity, thereby excluding a thioester intermediate. We next evaluated candidate 2, in which Acac‐alkyne may undergo transformation to Cro‐alkyne, followed by Michael addition to cysteine residues (Figure 3b). If it is the case, we postulated that the corresponding alkyne‐containing mimic of crotonate (Cro‐alkyne)^[^ ^18^ ^]^ could also form the same Cys + 267 modification. To test this, we synthesized Cro‐alkyne and performed the TOP‐ABPP assay to compare its labeling profile with that of Acac‐alkyne (Figure 3c). Among 133 peptides modified by Cro‐alkyne at Cys + 267, 24 sites exhibited co‐elution and matched MS2 fragmentation patterns with peptides labeled by Acac‐alkyne, indicating shared modification sites and suggesting overlapping reactivity profiles between the two probes at these positions (Figure S3c). Taking PRDX3 C229 as an example, the modified peptide (AFQYVETHGE‐VCPANWTPDSPTIKPSPAASK) showed the same retention times (Figure 3d) and fragmentation patterns in Acac‐alkyne and Cro‐alkyne groups (Figure S3d). Collectively, these results support candidate 2 as the novel cysteine modification structure, suggesting Acac‐alkyne can be converted to Cro‐alkyne, leading to Cys + 267 modification through Michael addition.

### Confirmation of Endogenous Cysteine Modification by Co‐Elution Assay with Standard Peptides

**Figure 4:**
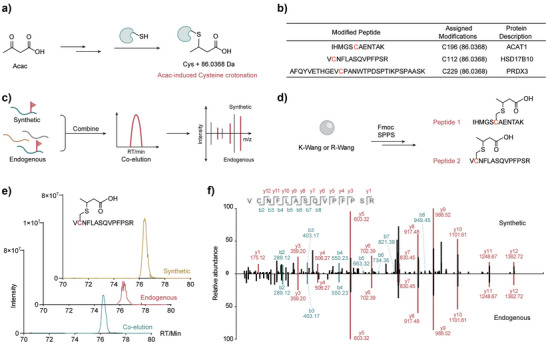
Identification of the endogenous cysteine modification by standard peptides co‐elution assay. a) Acac is proposed to induce Ccr. b) Ccr‐modified peptides derived from ACAT1 C196, HSD17B10 C112, and PRDX3 C229 were identified by PRM scanning. c) Validation of the authenticity of the modified peptides by Co‐Elution Assay. d) Synthesis of two modified peptides IHMGSCcrAENTAK and VCcrNFLASQVPFPSR by SPPS. e) The extracted ion chromatogram corresponding to Ccr modification of HSD17B10 C112 (VCcrNFLASQVPFPSR). f) MS2 spectra of the Ccr modification at HSD17B10 C112 (VCcrNFLASQVPFPSR).

After confirming the conversion of Acac‐alkyne to Cro‐alkyne, we hypothesized that endogenous Acac may also undergo a similar transformation to generate a crotonate intermediate, resulting in a + 86.0368 Da mass shift to cysteine residue (Figure 4a). We designated this modification as cysteine crotonation (Ccr). To test whether Acac treatment could induce Ccr under physiological conditions, we treated the cells with 10 mM Acac for 24 hours, isolated mitochondria and prepared samples for targeted detection using a PRM inclusion list identified by Acac‐alkyne metabolic labeling (Figure S4a). As a result, we detected three Ccr‐modified peptides derived from ACAT1 C196, HSD17B10 C112, and PRDX3 C229 (Figure 4b).

To ultimately validate the authenticity of the modified peptides, we performed the co‐elution experiment with synthetic peptides and Acac‐treated mitochondrial samples (Figure 4c), which is widely used as the gold standard to verify the structure of new modifications. ^[^ ^27^, ^28^ ^]^ We selected to synthesize two modified peptides IHMGSCcrAENTAK and VCcrNFLASQVPFPSR via solid‐phase peptide synthesis (SPPS) and conducted co‐elution experiments to confirm the identity of the endogenous peptides (Figure 4d). Briefly, K‐Wang or R‐Wang resin was coupled with C terminal amino acids and then reacted with Fmoc‐Cys(S‐Tmp)‐OH. ^[^ ^29^ ^]^ After Tmp de‐protection by DTT, the resin was treated with *tert*‐butyl crotonate and then subjected to conventional SPPS to complete the whole sequence with Ccr modification. To further confirm the crotonation occurs via a thioether linkage, truncated synthetic peptide (CcrNFLA) from HSD17B10 was synthesized and the presence of a thioether linkage was confirmed by NMR, demonstrating the reliability of the synthetic strategy. In addition, MS/MS analysis of two Ccr‐modified peptides, together with the high‐resolution MS/MS analysis *tert*‐butyl‐protected peptide (Figure S12), provided mutually reinforcing evidence that crotonation proceeds via a stable thioether linkage. The co‐elution assay with mitochondrial samples and synthetic peptides showed that the synthetic standard VCcrNFLASQVPFPSR (Figure 4e,f) and IHMGSCcrAENTAK (Figure S4c, d) have the same retention time and MS2 fragmentation patterns as the endogenously Ccr‐modified peptides. Notably, the IHMGSCcrAENTAK peptide consistently revealed the same ion peaks but two distinct retention times, suggesting that the Ccr modification exists as both *R*‐ and *S*‐stereoisomers. This observation supports that the Michael addition step involved in Ccr formation proceeds without stereoselectivity. Collectively, these results confirm that endogenous Ccr modification exists in cells in response to Acac treatment.

### Defining the Metabolic Pathway for Ccr Formation

**Figure 5:**
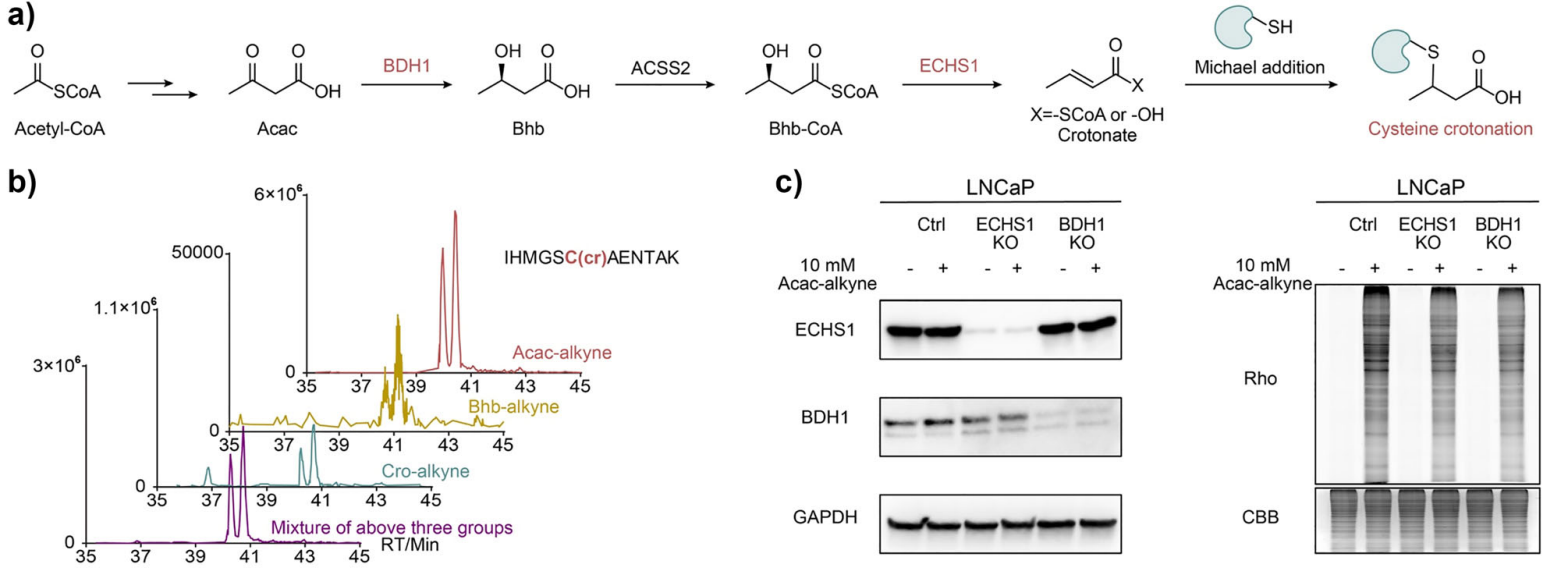
Exploration of the metabolic pathway for Ccr formation. a) Acac is expected to be first reduced to Bhb, which may subsequently undergo dehydration to generate crotonyl intermediate, then induce cysteine crotonation (Ccr). b) The extracted ion chromatogram corresponding to ACAT1 C196 in Acac‐alkyne, Bhb‐alkyne, Cro‐alkyne and mixture groups. c) BDH1 and ECHS1 KO cell lines exhibited reduced fluorescence signals compared to WT cell line, indicating a suppression of Ccr modification. The corresponding uncropped original gels/blots are shown in Figure S10 within the Supporting Information.

To investigate the endogenous origin of Ccr, we next examined the potential biochemical pathway leading to its formation. Chemically, Acac is expected to be reduced to Bhb first, which may subsequently undergo dehydration to generate the crotonyl intermediate (Figure 5a). This raises a key mechanistic question: if this pathway is operative, could all Acac, Bhb and crotonate serve as precursors for Ccr? To test this hypothesis, we showed that both Cro‐alkyne and crotonate can undergo spontaneous, non‐enzymatic Michael addition reactions with glutathione (GSH) (Figure S5a,b), supporting the possibility that crotonyl intermediates can non‐enzymatically react with cysteine residues. To next test whether Bhb can induce Ccr formation, we synthesized an alkyne‐containing Bhb derivative (Bhb‐alkyne) probe (Figure S5c) and performed a series of co‐elution experiments using all three probes. These experiments confirmed that Acac‐, Bhb‐, and Cro‐derived probes can modify the same cysteine sites at ACAT1 C196, PRDX3 C229, and HSD17B10 C112 (Figures 5b and S5d,e) and share reliable MS2 fragmentation spectra (Figure S5c), supporting Bhb as a viable Ccr precursor and showing the plausibility of a shared metabolic pathway with Acac.

Given that the conversion from Acac to crotonyl intermediates likely involves enzymatic steps, we further investigated the role of key metabolic enzymes. 3‐Hydroxybutyrate dehydrogenase 1 (BDH1)^[^ ^30^ ^]^ is known to catalyze the reduction of Acac to Bhb, while Enoyl‐CoA Hydratase, Short Chain 1 (ECHS1)^[^ ^31^ ^]^ is implicated in the sub‐sequent dehydration step to form the crotonate intermediate (Figure 5a). To evaluate their involvement, we generated BDH1 and ECHS1 CRISPR knockout (KO) cell lines and performed metabolic labeling using Acac‐alkyne (Figure 5c, S10). Notably, both KO cell lines exhibited reduced fluorescence signals compared to WT control, indicating a suppression of Ccr modification formation. Together, these findings support a model in which Acac is converted to Bhb and then to a crotonyl intermediate that undergoes Michael addition with cysteine residues to form Ccr, with BDH1 and ECHS1 serving as critical enzymatic regulators of this pathway.

### Ccr at Cys229 of PRDX3 Modulates Redox Balance

**Figure 6:**
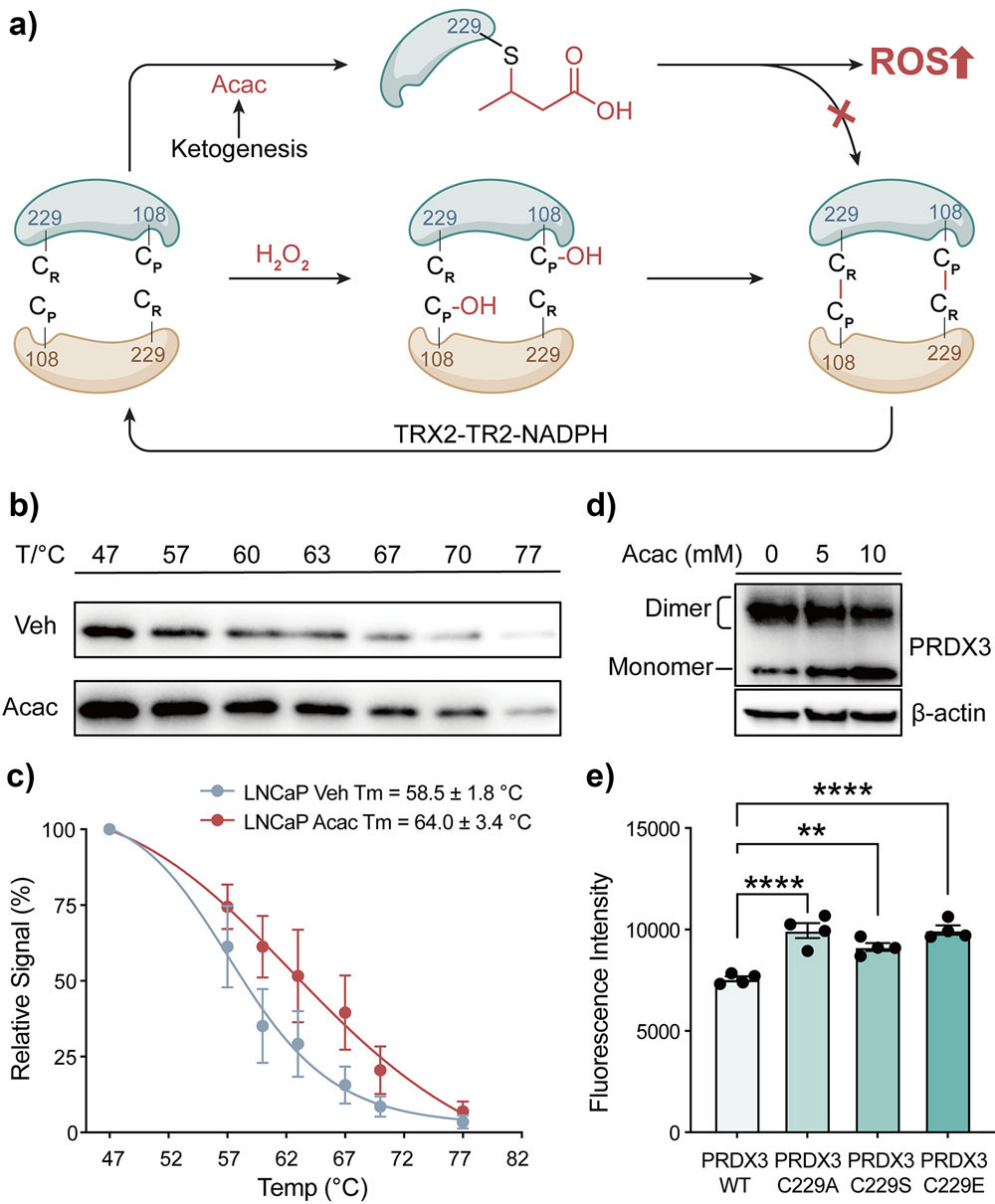
Ketogenesis‐induced cysteine crotonation at Cys229 of PRDX3 impairs redox activity and promotes oxidative stress. a) Schematic showing that Acac metabolism induces PRDX3 C229cr, blocking disulfide bond formation with Cys108 and impairing ROS detoxification. b) CETSA of PRDX3 protein in LNCaP cells with or without 10 mM Acac, assessed by western blotting. c) Thermal shift curves of PRDX3 were derived from (b), showing increased Tm after Acac treatment (*n* = 3; mean ± SEM). d) Non‐reducing Western blot showed that Acac inhibited PRDX3 dimerization via disulfide bonds in a dose‐dependent manner. e) Flow cytometry of ROS levels using CM‐H2DCFDA in LNCaP cells overexpressing PRDX3 WT or cysteine mutants after Acac treatment. Mutants showed elevated ROS (*n* = 4; mean ± SEM; ***p < 0.001, ****p < 0.0001, one‐way ANOVA). The corresponding uncropped original gels/blots are shown in Figure S11 within the Supporting Information.

To further investigate the cellular impact of Acac‐induced Ccr, we focused our analysis on PRDX3. PRDX3 is a mitochondrial peroxiredoxin that detoxifies ROS by undergoing redox cycling through the formation of disulfide bonds between its catalytic cysteine residues at C108 (peroxidatic cysteine, C_P_) and C229 (resolving cysteine, C_R_). From our proteomics data, we identified C229, a key resolving cysteine in PRDX3's catalytic cycle, as a site of Ccr. Furthermore, we performed proteomic study at lower Acac concentration (0.3 mM), modification of C229 was still detected (Figure S6a). We hypothesized that Ccr at C229 would interfere with disulfide bond formation, disrupt dimerization,^[^ ^32^ ^]^ and impair PRDX3's ROS‐scavenging function (Figure 6a). From the cellular thermal stability assay (CETSA), we observed that Acac treatment increased the melting temperature (Tm) of PRDX3, indicating that Ccr enhances PRDX3 protein stability (Figures 6b,c and S6b, S11). Furthermore, nonreducing immunoblots showed that Acac treatment reduced PRDX3 dimer formation and increased monomer levels (Figures 6d and S11), indicating the Ccr modification could disrupt PRDX3 dimerization. Finally, Acac exposure led to elevated ROS levels in LNCaP cells, which could be rescued by overexpression of WT PRDX3 but not by C229 mutants. Notably, the C229E mutant, a mimic of Ccr, phenocopied C229A and C229S in their inability to suppress ROS (Figures 6e and S6c–e). These results supported our hypothesis that crotonation at the C229 impairs the antioxidant function of PRDX3 and contributes to redox imbalance.

## Discussion

Ketone bodies are increasingly produced by the liver during periods of low carbohydrate availability (e.g., fasting, exercise, or ketogenic diets). In addition to serving as energy substrates, ketone bodies function as metabolic regulators that support brain activity, suppress inflammation, modulate immune responses, fuel the heart efficiently and reshape cancer metabolism.^[^ ^33^, ^34^, ^35^, ^36^, ^37^ ^]^ Recent studies have identified Kbhb and Kacac as PTMs mediated by ketone bodies, linking them to diverse physiological and pathological processes.^[^ ^38^ ^]^ In our study, we discovered that ketone bodies can also induce previously uncharacterized cysteine modifications, namely Cys + 427 and Cys + 267.

We structurally confirmed the Cys + 267 modification through both probe‐based labeling and synthetic peptide co‐elution assays, revealing its origin from the metabolic conversion of Acac into a crotonyl intermediate. Interestingly, a recent study by the Wang group reported carboxyalkylation modifications derived from long‐chain fatty acid metabolism using the IsoSTAR platform.^[^ ^39^ ^]^ While the modification is related, the cysteine crotonation reported here appears to be distinct in both its metabolic precursor and biosynthetic pathway. Acac is derived from ketogenesis and mainly produced in human liver and then circulates to extrahepatic tissues. We showed Acac is metabolized to Bhb first and subsequently to crotonyl intermediates that covalently react with cysteine thiols to generate Ccr, with BDH1 and ECHS1 as key enzymes for this transformation. During starvation, Acac and Bhb are frequently reported at millimolar levels;^[^ ^1^, ^2^, ^3^ ^]^ whereas during diabetic ketoacidosis, Acac can reach ∼6 mM, and Bhb can reach ∼18 mM in blood.^[^ ^4^ ^]^ At sites of ketone body production, Acac local concentrations may be even higher. These considerations motivated our use of millimolar‐range Acac and Acac‐alkyne to approximate physiologically relevant exposures. Moreover, we found that Bhb also induces Ccr, supporting the generality of ketone body–induced Ccr.

Despite these advances, several aspects of the study require further investigation. The precise structure of the Cys + 427 modification remains unresolved and needs further confirmation. These discoveries expand the repertoire of cysteine modifications.^[^ ^40^ ^]^ Moreover, although CRISPR‐mediated knockout of ECHS1 and BDH1 significantly reduced labeling, in‐gel fluorescence signals were still observed, suggesting that compensatory metabolic pathways may also contribute to Ccr formation that needs to be further investigated.

Our findings reveal that ketone metabolism can drive novel cysteine modifications that may fundamentally reshape our understanding of ketogenesis and its regulatory functions in biology. Ketone bodies and their downstream metabolites possess chemically reactive functional groups, such as ketones/aldehydes, α,β‐unsaturated carboxylic acids, and α‐hydrogens adjacent to carbonyls, that can engage in diverse covalent interactions with proteins. These functionalities offer multiple avenues for PTM, either through enzymatic transfer or nonenzymatic reactivity. While lysine acylations such as Kbhb and Kacac have been the primary focus,^[^ ^41^ ^]^ our findings suggest that other nucleophilic residues, including cysteine, may also serve as modification sites under ketogenic conditions. Given the diversity of ketone‐derived metabolites and their metabolic flux in cells, it is likely that additional, currently unrecognized PTMs exist. These findings highlight the need to systematically explore the PTM landscape driven by ketone metabolism and expand our chemical understanding of metabolite‐protein interactions. Unlike lysine crotonylation^[^ ^42^ ^]^ or cysteine acylation,^[^ ^43^, ^44^ ^]^ cysteine alkynation^[^ ^45^ ^]^ represents a novel type of PTM involving a thioether bond, whose mechanism, regulation, and function remain largely unexplored. Although our identified Ccr arises from non‐enzymatic Michael addition, the inherent reversibility of this reaction suggests the potential for dynamic regulation.^[^ ^46^ ^]^ Michael addition has been reported to induce several cysteine thioether linkages, including cysteine succination,^[^ ^40^, ^47^ ^]^ fumaration,^[^ ^48^ ^]^ and itaconation.^[^ ^49^ ^]^ Given a similar thioether PTM, is catalyzed by cystathionine beta synthase, we remain open to the existence of yet‐undiscovered Ccr writers, readers, and erasers.^[^ ^50^ ^]^

Interestingly, the Ccr modification identified in this study was exclusively localized to mitochondrial proteins, particularly those involved in redox regulation and energy metabolism. This compartmental specificity implies that the mitochondrial microenvironment, characterized by high thiol content, ROS, and dynamic metabolic flux, may uniquely favor cysteine modification by ketone‐derived intermediates. Enzymes such as PRDX3, ACAT1, and HSD17B10, central to detoxification, ketone utilization, and β‐oxidation,^[^ ^51^, ^52^, ^53^ ^]^ were among the Ccr‐modified targets. The spatial restriction of this modification points to a functional link between Ccr and sophisticated mitochondrial redox regulation, raising intriguing possibilities for its role in modulating mitochondrial signaling, stress response, and the metabolic‐redox interface. Our current study showed that Ccr at PRDX3 C229 impairs its disulfide bond formation and redox activity, resulting in elevated ROS levels. This is consistent with previous studies showing that dynamic S‐acylation of PRDX family enzymes modulates ROS stress and signaling and further provides an additional layer of PRDX regulation through cysteine‐based PTM.^[^ ^54^ ^]^ In addition to PRDX3, our methods identified over 170 additional potential Ccr sites. Elucidating whether these sites are endogenously related to the biological effects of ketone bodies represents an important direction for future research.

## Supporting Information

The Supporting Information includes experimental procedures, Figures S1–S12 and data for probe synthesis, chemical proteomics, biological assays, and bioinformatics analysis are provided in the Electronic Supplementary Information (ESI). (Table S1) Site‐specific identification of cysteine modifications enabled by the Acac‐alkyne probe.

## Conflict of Interests

The authors declare no conflict of interest.

## Supporting information



Supporting Information

Supporting Information

## Data Availability

The mass spectrometry proteomics data have been deposited to the ProteomeXchange Consortium via the iProX partner repository with the dataset identifier PXD066371.
