# Supplementary material for: Chemical Proteomics Identifies Ketogenesis‐Mediated Cysteine Modifications Regulating Redox Function
Source: Angew Chem Int Ed Engl. 2026 Jan 16;65(14):e19830. doi: 10.1002/anie.202519830 (PMC13023691; doi:10.1002/anie.202519830)
Supplement: Supplementary file 1 — Supporting Information [file ANIE-65-e19830-s001.docx]

Supporting Information

Chemical Proteomics Identifies Ketogenesis-Mediated Cysteine Modifications Regulating Redox Function

Yuan-Fei Zhou^1^, Ling Zhang^1^, Zhuoyi L. Niu^1^, Xin Wang^2^, Alejandro Storper^1^, Ryan Hunt^1^, Yingming Zhao^3^, Nima Sharifi^1^, Zhipeng A. Wang^1，^*

1. Desai Sethi Urology Institute & Sylvester Comprehensive Cancer Center, University

of Miami Miller School of Medicine, Miami, FL 33136, USA

2. Department of Microbiology and Cell Science, Institute of Food and Agricultural

Sciences, University of Florida, Gainesville, FL 32611, USA

3. Ben May Department for Cancer Research, The University of Chicago, Chicago, IL

60637, USA

* E-mail: zaw29@miami.edu

**Contents**

1. **Supplementary figures S3**
2. **Organic synthesis materials and methods S14**
3. **Biological experiments materials and methods S18**
4. **Chemical proteomics study S19**
5. **Supplementary tables S21**
6. **Characterization of the compounds S21**
7. **References S31**
8. **Supplementary figures**

**
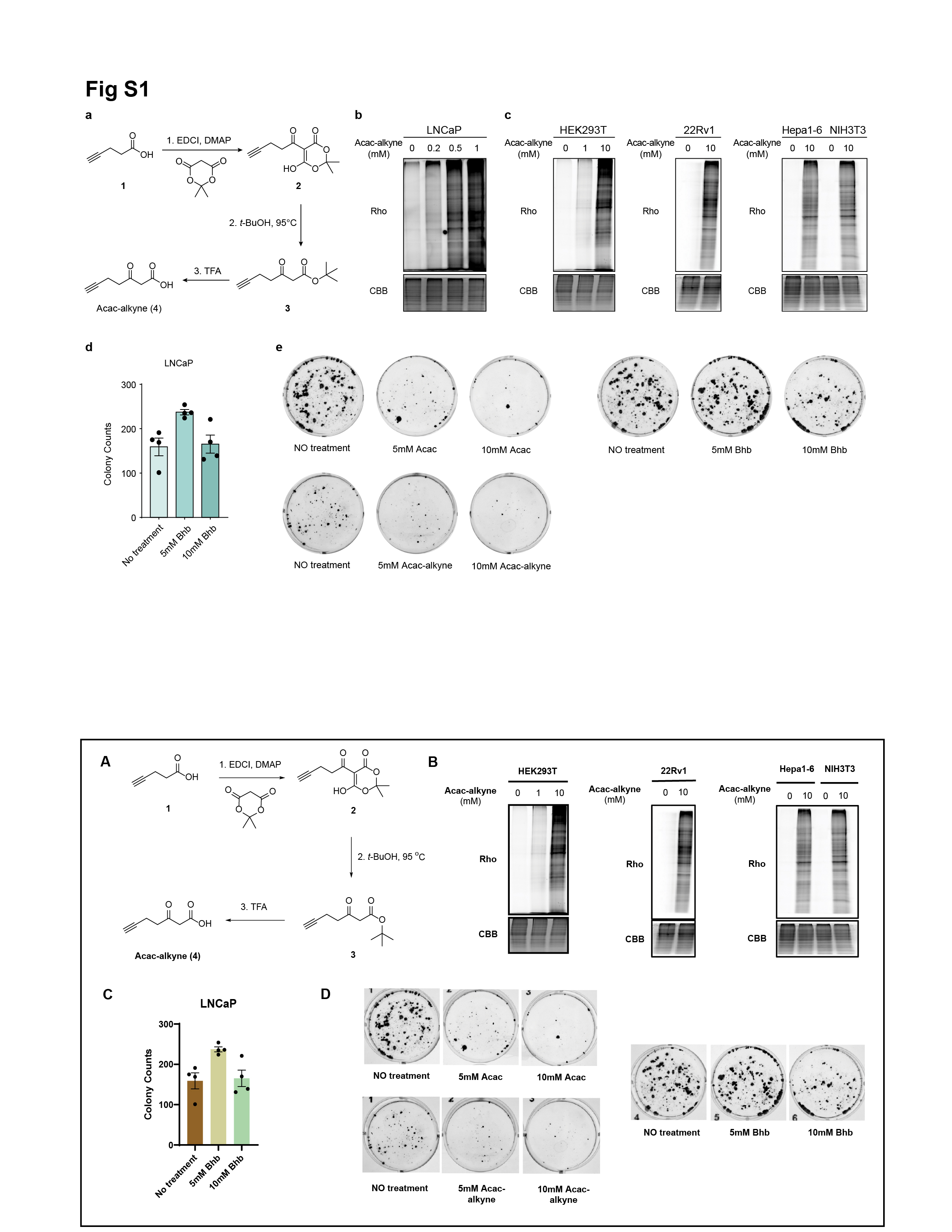
**

**Figure S1.** (a) Chemical synthesis of Acac-alkyne. (b) LNCaP cells were labeled with increasing concentrations of Acac-alkyne, showing a dose-dependent manner. Coomassie Brilliant Blue (CBB) staining was used as a loading control. (c) In-gel fluorescence of Acac-alkyne probe in HEK293T, 22Rv1, Hepa1-6 and NIH3T3 cell lines. (d) Bhb did not show colony-suppressive effects. (e) Representative images from the LNCaP cell line colony formation assay. The corresponding uncropped Rho-stained and corresponding CBB images are shown in Figure S8 within the Supporting Information.


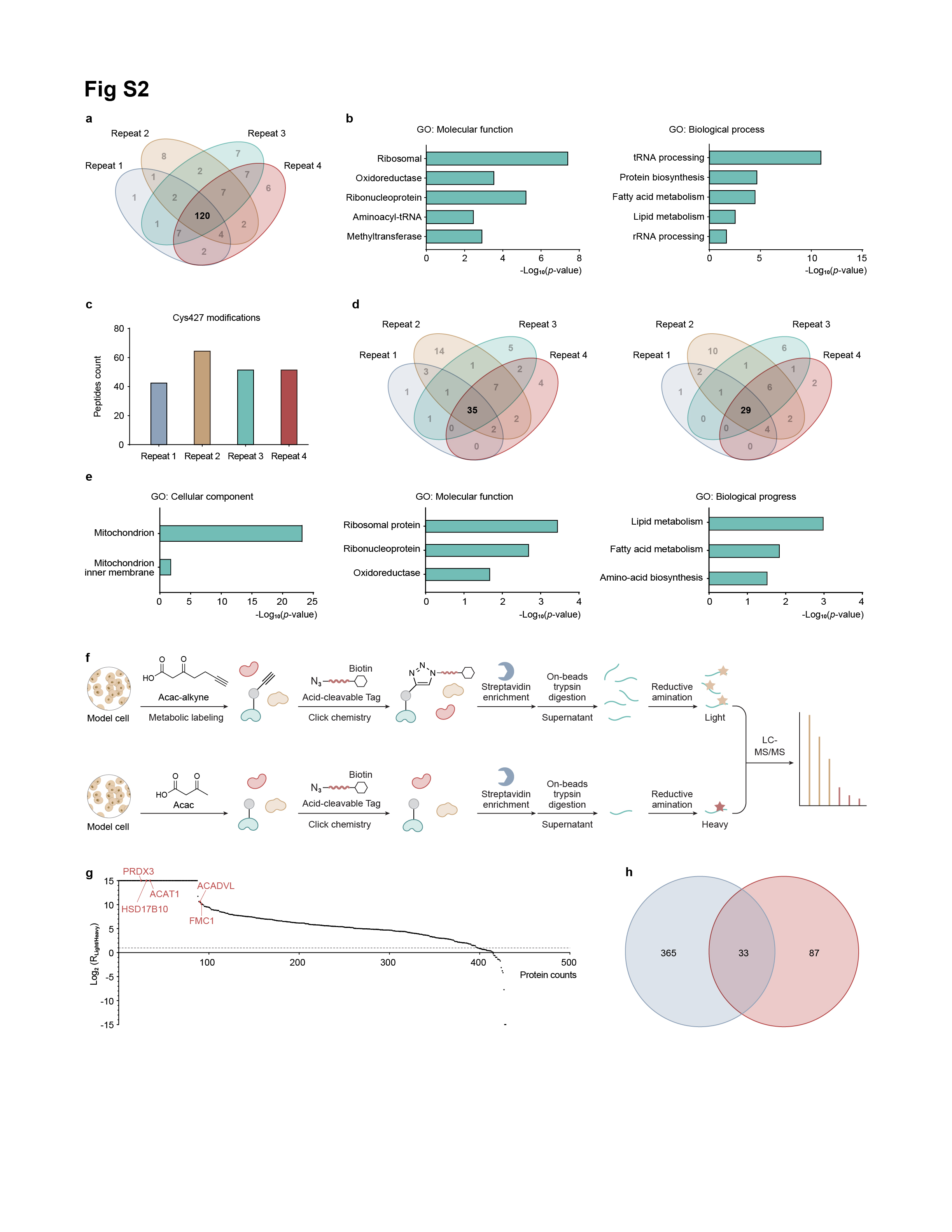


**Figure S2**. (a) The chemical proteomics data indicated 120 distinct proteins with Cys+267 modification across four replicates. (b) GO enrichment analysis of Cys+267-modified proteins in the categories of molecular function and biological process. (c, d) The chemical proteomics data showed 35 peptides containing Cys+427 modification, mapping to 29 proteins. (e) GO enrichment analysis of Cys+427-modified proteins in the categories of cellular component, molecular function and biological process. (f) Schematic workflow for quantitative identification of Acac-alkyne enrichment effect of Acac-modified proteins. Cells were metabolically labeled with Acac-alkyne or Acac, followed by click conjugation to an acid-cleavable biotin tag, streptavidin enrichment, on-bead digestion, reductive dimethyl labeling (light/heavy), and LC–MS/MS analysis. (g) Rank-ordered waterfall plot showing log_2_(light/heavy) ratios of quantified proteins from the quantitative experiment. The dashed line indicates the enrichment threshold (log₂ L/H = 1). Protein examples with strong enrichment upon Acac-alkyne labeling are highlighted, including PRDX3, ACAT1, HSD17B10, ACADVL, and FMC1. (h) Venn diagram comparing proteins quantified by quantitative experiment (left) with proteins for which Cys+267 modification sites were directly identified by site-specific TOP-ABPP (right). The overlap represents proteins that are both quantitatively enriched and contain confidently assigned Cys+267 modification sites.
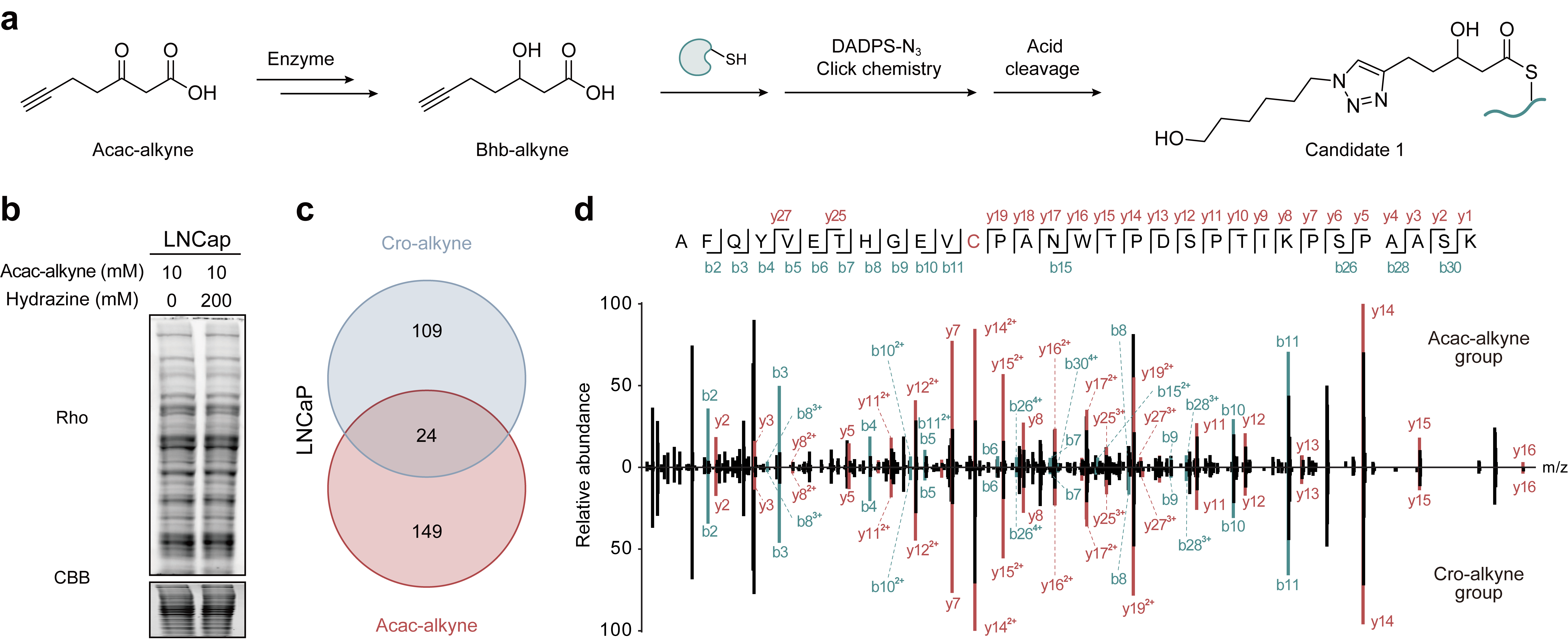


**Figure S3.** (a) Proposed candidate 1 to form Cys+267 modification. (b) In-gel samples treated with 0.2 M hydrazine showed no detectable change in fluorescence intensity. (c) 133 sites were identified to have Cys+267 modification in Cro-alkyne group, sharing 24 common sites with that of Acac-alkyne. (d) MS2 spectra of the Cys+267 modification at PRDX3 C229 (AFQYVETHGEV**C**PANWTPDSPTIKPSPAASK). The corresponding uncropped Rho-stained and corresponding CBB images are shown in Figure S9 within the Supporting Information.





**Figure S4**. (a) Mitochondrial extracts were analyzed using PRM with an inclusion list incorporating modification site information. (b) Detailed procedures for the synthesis of the two modified peptides. (c) The extracted ion chromatogram corresponding to Ccr modification of ACAT1 C126 (IHMGSCcrAENTAK). (d) MS2 spectra of the Ccr modification at ACAT1 C126 (IHMGSCcrAENTAK).

**
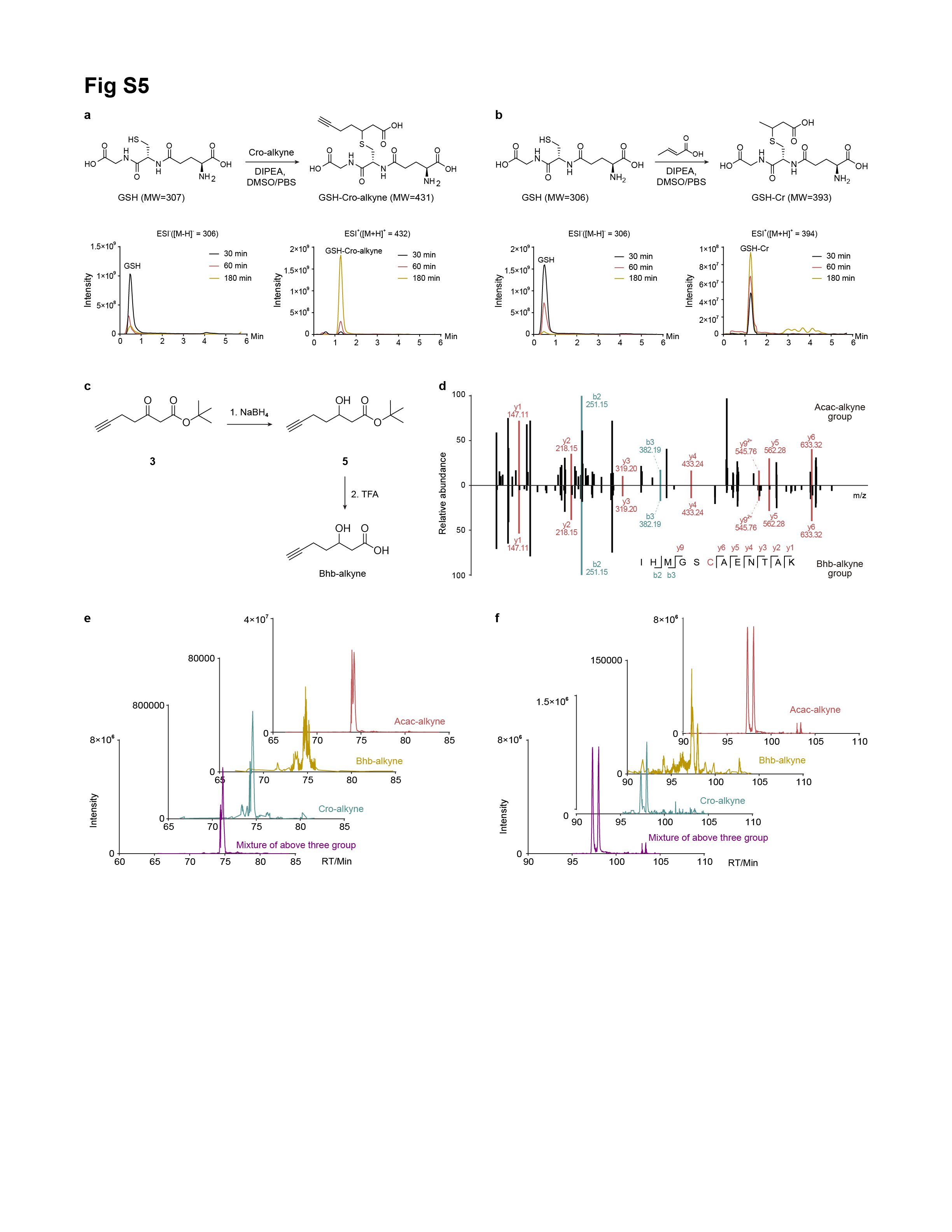
**

**Figure S5**. Monitoring the non-enzymatic Michael addition reaction between cro-alkyne (a) or crotonate (b) and GSH in different time points. (c) Synthesis of Bhb-alkyne. (d) MS2 spectrum of the probe modification at ACAT1 C126 (IHMGSCcrAENTAK). (e, f) The extracted ion chromatogram corresponding to PRDX3 C229 and HSD17B10 C112 in Acac-alkyne, Bhb-alkyne, Cro-alkyne and mixture groups, respectively.

**
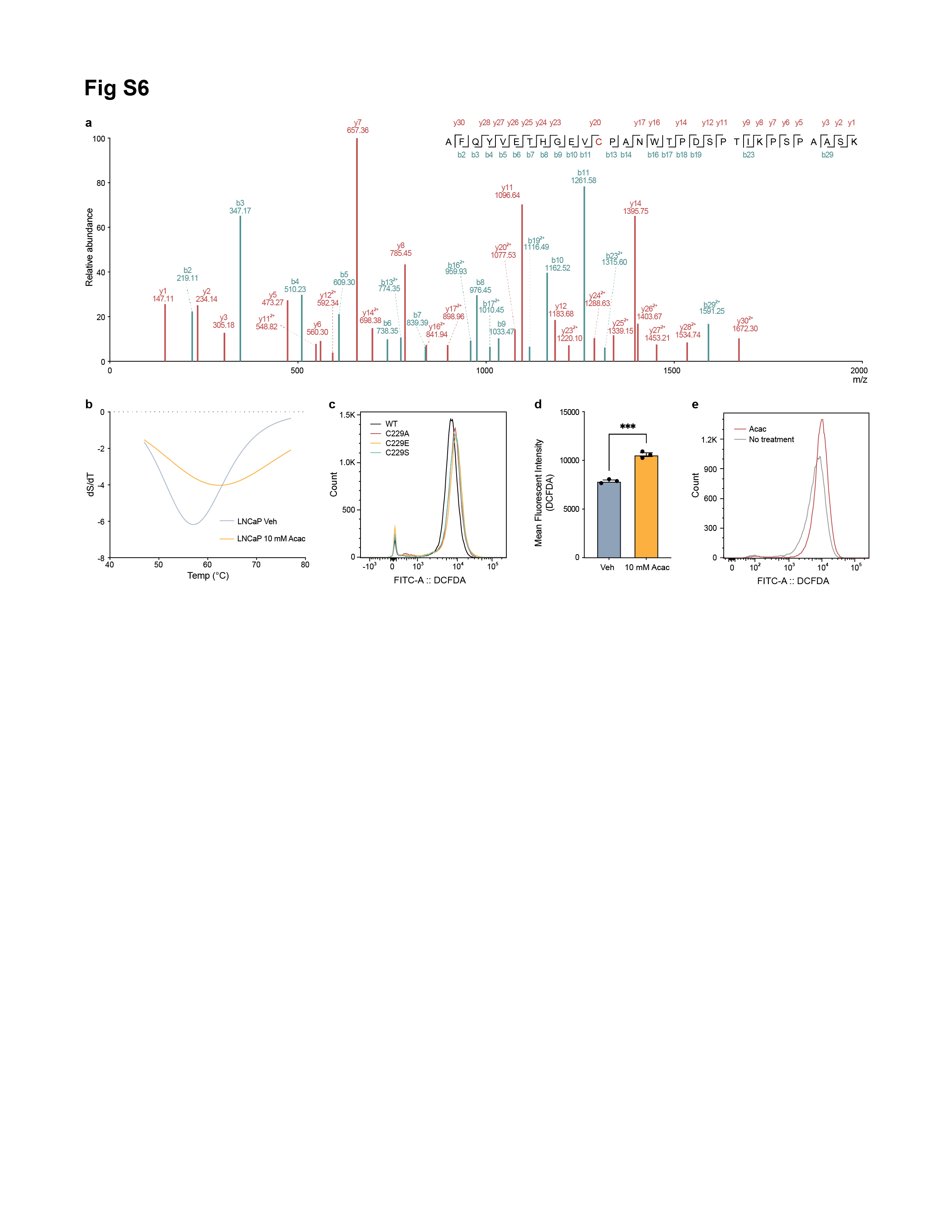
**

**Figure S6**. Acetoacetate induced PRDX3 destabilization and ROS accumulation. (a) PRM detection of PRDX3 peptide with C229cr under low Acac treatment condition (0.3 mM), demonstrating that Ccr is detectable at sub-millimolar Acac concentrations. (b) Representative derivative melting curves (dS/dT) of PRDX3 protein in LNCaP cells treated with vehicle or 10 mM Acac, assessed by thermal shift assay, showing increased thermal stability after Acac treatment. (c) Flow cytometry histograms of ROS levels (DCFDA staining) in LNCaP cells overexpressing PRDX3 WT or indicated cysteine point mutants (C221A, C229S, C229E). (d) Quantification of ROS levels in LNCaP cells treated with vehicle or 10 mM Acac for 24 h (n = 3; mean ± SEM; ***p < 0.001, unpaired t-test). (e) Representative histogram showing increased ROS in LNCaP cells treated with 10 mM Acac compared to untreated control (DCFDA staining).


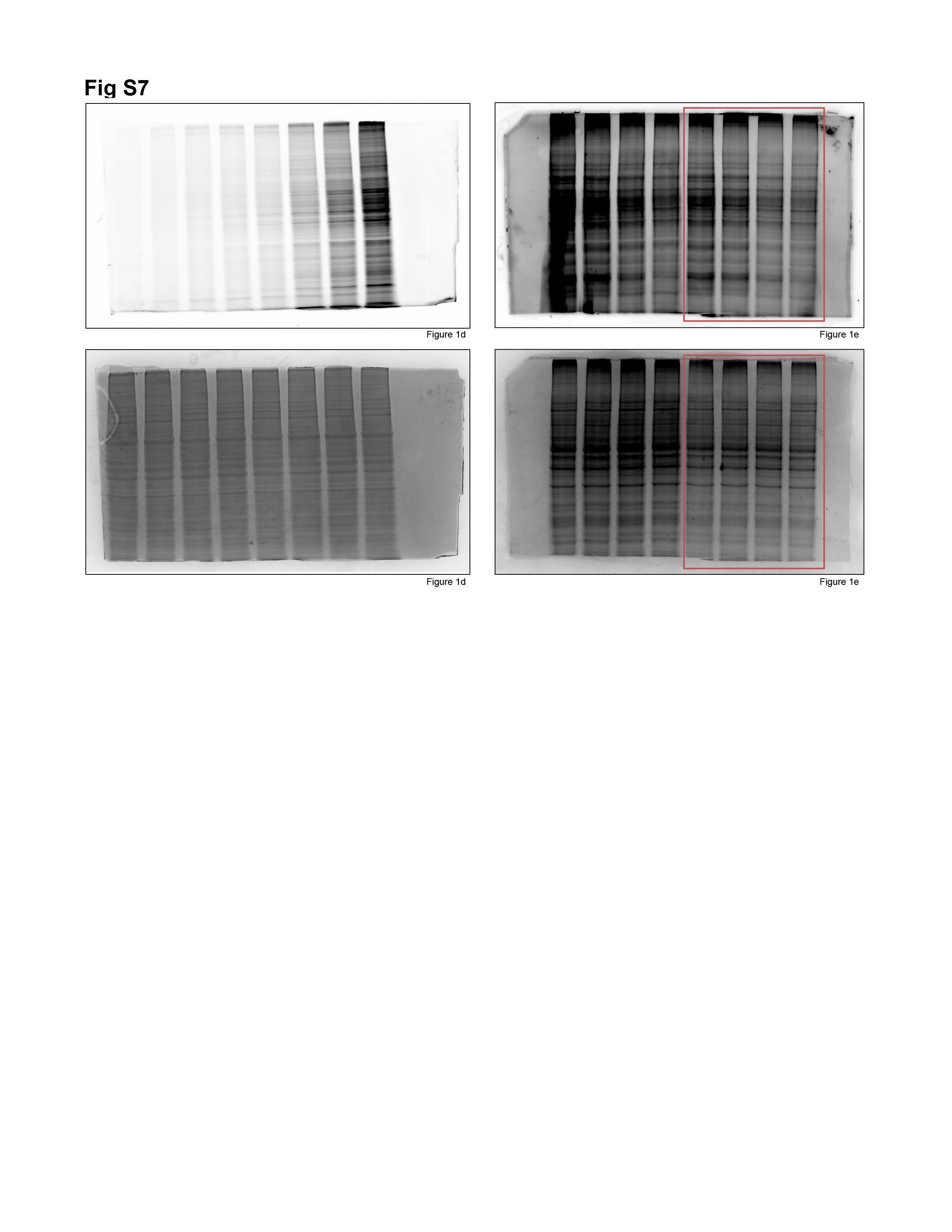


**Figure S7**. Uncropped blot for Figure 1d, e in the manuscript.
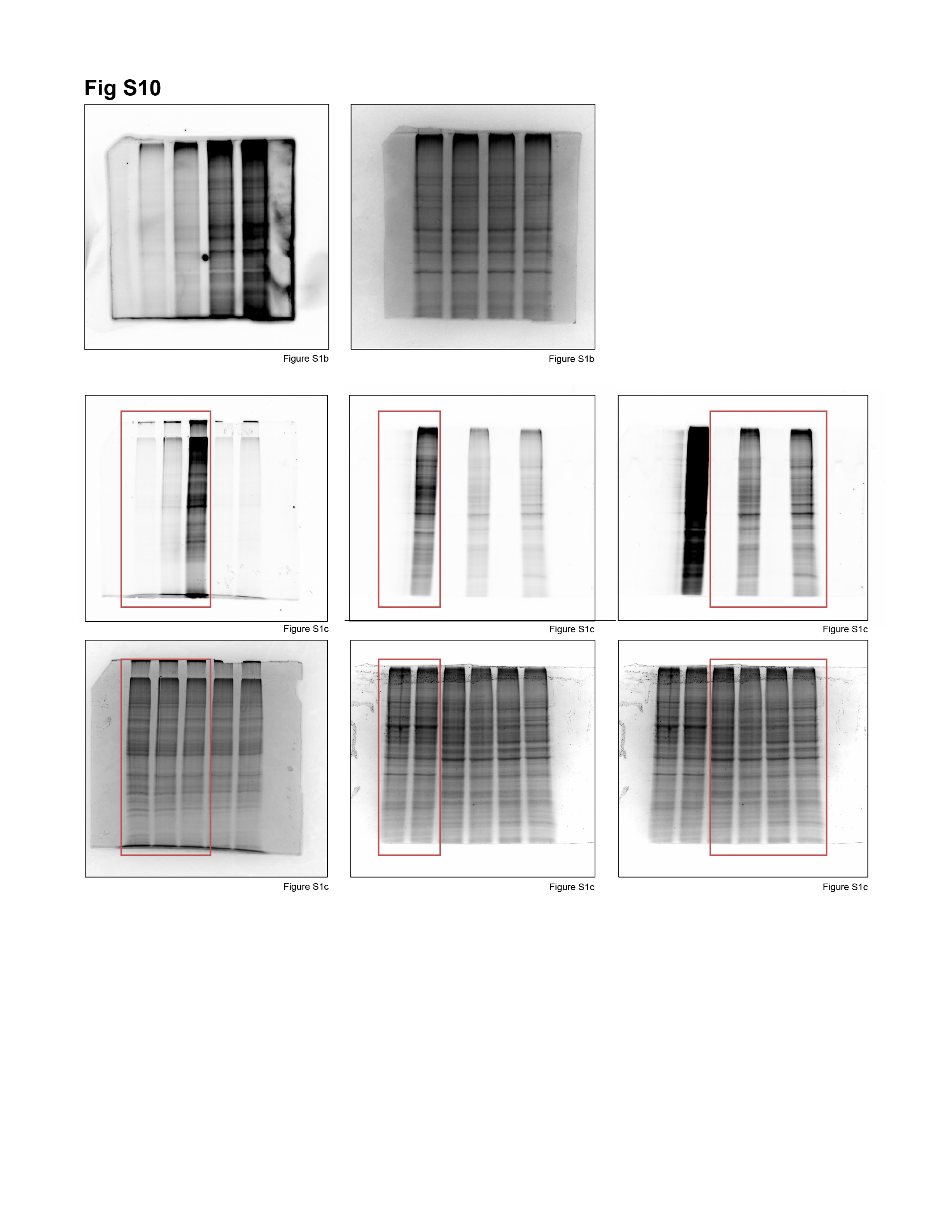


**Figure S8**. Uncropped blot for Figure S1b, c in the manuscript.


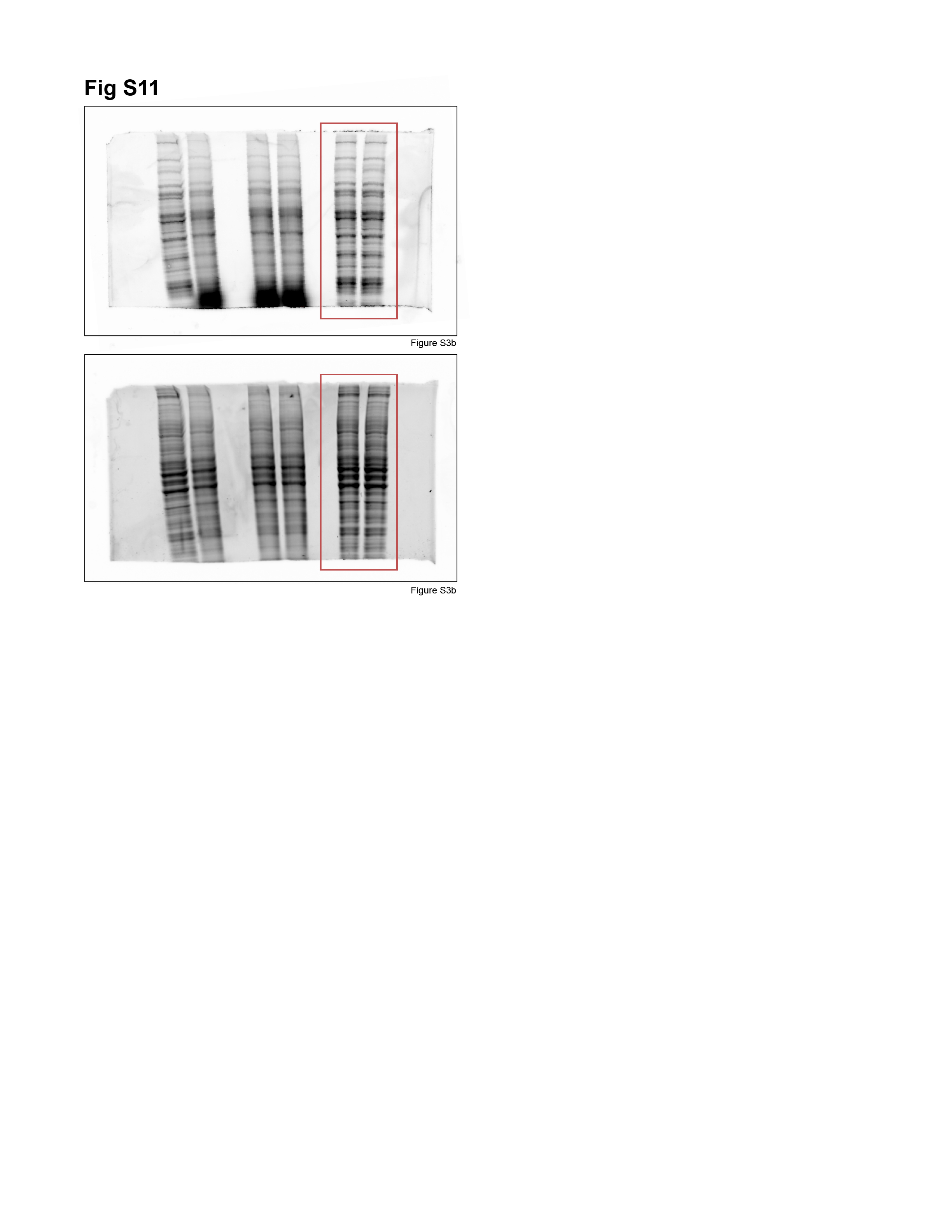


**Figure S9**. Uncropped blot for Figure S3b in the manuscript.


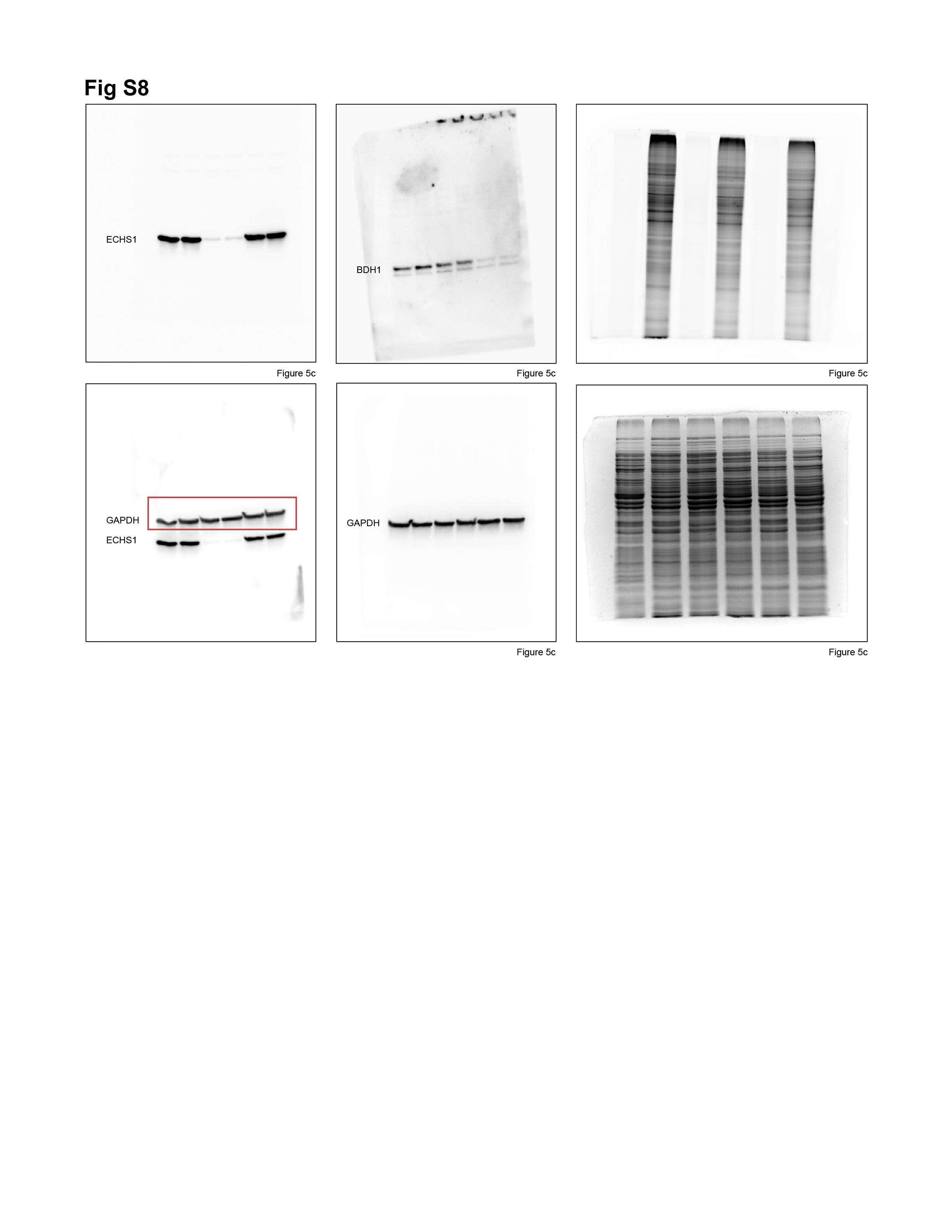


**Figure S10**. Uncropped blot for Figure 5c in the manuscript.

**
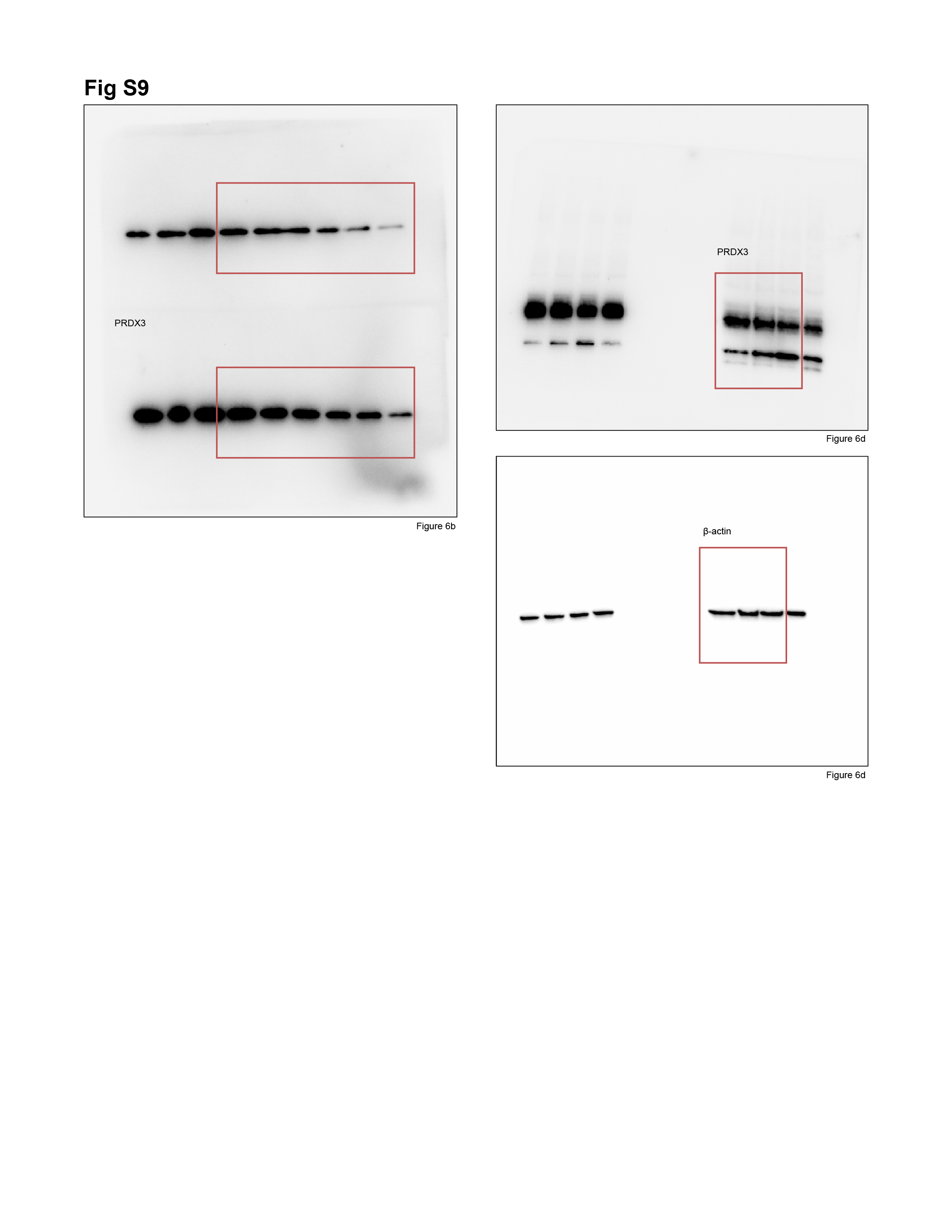
**

**Figure S11**. Uncropped blot for Figure 6b, d in the manuscript.

1. **Organic synthesis materials and methods**

**2.1 General Information**

All reactions were carried out under an argon atmosphere with dry solvents under anhydrous conditions, unless otherwise noted. Reagents were purchased at the highest commercial quality and used without further purification, unless otherwise stated. Solvents for chromatography were used as supplied by Merck chemicals. Reactions were monitored by thin layer chromatography (TLC) carried out on 0.2 mm Merck gel plates (60F-254) using UV light as visualizing agent and aqueous ammonium cerium nitrate/ammonium molybdate as developing agent. Merck silica gel (60, particle size 0.040–0.063mm) was used for flash column chromatography. NMR spectra were recorded on Bruker Advance 500 or 800 MHz. The spectra were calibrated by using residual undeuterated solvents (for ^1^H NMR) and deuterated solvents (for ^13^C NMR) as internal references: chloroform (*δ*_H_ = 7.26 ppm) and CDCl_3_ (*δ*_C_ = 77.16 ppm). The following abbreviations are used to designate multiplicities: s = singlet, d = doublet, t = triplet, q = quartet, m = multiplet, quint = quintet, br = broad. High-resolution mass spectra (HR-FT-MS) were recorded on Bruker impact II Q-TOF MS.

**2.2 Acac-alkyne and Bhb-alkyne probe synthesis**

**Synthesis of compound 2**

The compound **1** (4-Pentynoic acid, 20 mmol, 2.00 g), 2,2-dimethyl-1,3-dioxane-4,6-dione (30 mmol, 4.41 g), 1-Ethyl-3-(3-dimethylaminopropyl)carbodiimide (EDCI) (40 mmol, 7.82 g), and 4-Dimethylaminopyridine (DMAP) (40 mmol, 4.98 g) were dissolved in 20 mL of dichloromethane (DCM) and stirred at room temperature for 3 hours. The reaction was quenched by the addition of 50 mL of saturated aqueous sodium carbonate. The aqueous phase was extracted with DCM (3 × 100 mL). The combined organic layers were dried over anhydrous sodium sulfate, filtered, and concentrated under reduced pressure. The crude product was purified by flash column chromatography (DCM: acetone = 10:1, v/v) and further concentrated under reduced pressure to yield compound **2** as a yellow solid (2.617 g, 58% yield). ^1^H NMR (500 MHz, Chloroform-*d*) δ 3.30 (m, 2H), 2.59 (m, 2H), 1.95 (s, 1H), 1.69 (s, 6H). ^13^C NMR (126 MHz, CDCl_3_) δ 195.0, 170.6, 160.3, 105.3, 92.1, 81.8, 69.8, 34.6, 27.0, 14.9 HR-ESI (m/z): [M - H]^-^ calcd for C_11_H_12_O_5_ 223.0606, found 223.0589.

**Synthesis of Compound 3**

Compound **2** (0.65 g, 2.90 mmol) was dissolved in *tert*-butanol (10 mL) and stirred at 95 °C in an oil bath for 4 hours. Upon completion, the solvent was removed under reduced pressure. The crude residue was purified by flash column chromatography (PE: EA = 20:1, v/v) to afford compound **3** as a colorless oil (0.31 g, 54.5% yield). ^1^H NMR (800 MHz, Chloroform-*d*) δ 3.35 (s, 2H), 2.78 (m, 2H), 2.45 (m, 2H), 1.93 (s, 1H), 1.45 (s, 9H). ^13^C NMR (201 MHz, CDCl_3_) δ 201.2, 166.3, 82.9, 82.4, 69.1, 50.7, 41.7, 28.2, 13.0. HR-ESI (m/z): [M + Na]^+^ calcd for C_11_H_16_O_3_Na 219.0997, found 219.0994.

**Synthesis of compound 4 (Acac-alkyne)**

Compound **3** (0.31 g, 1.58 mmol) was dissolved in dichloromethane (DCM, 2 mL), and trifluoroacetic acid (TFA, 1 mL) was added dropwise at room temperature. The reaction mixture was stirred for 2 hours and monitored by TLC. The solvent and excess TFA were then removed under reduced pressure to afford compound **4** as a gray solid (0.202 g, 91.2% yield). ^1^H NMR (500 MHz, Methanol-*d*) δ 3.24 (dt, *J* = 3.3, 1.6 Hz, 1H), 2.76 (t, *J* = 7.2 Hz, 2H), 2.36 (m, 2H), 2.35 (d, *J* = 2.7 Hz, 1H), 2.15 (t, *J* = 2.7 Hz, 1H). ^13^C NMR (126 MHz, MeOD) δ 203.6, 170.9, 83.8, 70.0, 49.0, 42.8, 13.6. HR-ESI (m/z): [M + H]^+^ calcd for C_7_H_8_O_3_ 163.0371, found 163.0362.

**Synthesis of compound 5**

Compound **3** (0.69 g, 3.52 mmol) was dissolved in methanol (5 mL) and cooled in an ice bath. Sodium borohydride (0.160 g, 4.22 mmol, 1.2 equiv) was added portionwise with stirring at 0 °C. The reaction was maintained at 0 °C and stirred for 1 hour. Acetone (30 mL) was then added to quench the reaction. The solvent was removed under reduced pressure to afford a crude oil, which was subsequently purified by flash column chromatography. The column was first eluted with pure dichloromethane (DCM), followed by a gradient of DCM: acetone (10:1, v/v), to yield compound **5** as a yellow oil (0.633 g, 90.7% yield). ^1^H NMR (800 MHz, Chloroform -*d*) δ 4.08 (m, 1H), 3.19 (d, *J* = 3.8 Hz, 1H), 2.41 (dd, *J* = 16.5, 3.0 Hz, 1H), 2.33 (m, 2H), 1.93 (t, *J* = 2.8 Hz, 1H), 1.67 (m, 1H), 1.61 (m, 1H), 1.44 (s, 9H). ^13^C NMR (201 MHz, CDCl_3_) δ 172.5, 84.1, 81.7, 68.9, 67.0, 42.3, 35.1, 28.3, 15.0. HR-ESI (m/z): [M + H]^+^ calcd for C_11_H_18_O_3_ 221.1154, found 221.1148.

**Synthesis of compound 6**

Compound **5** (0.633 g, 3.19 mmol) and TFA (1.5 mL) was added dropwise in DCM (3 mL) at room temperature. The reaction mixture was stirred for 2 hours. The solvent and excess TFA were then removed under reduced pressure to afford compound **6** as an orange solid (0.448 g, 98.8% yield). ^1^H NMR (500 MHz, Methanol-*d*) δ 4.01 (m, 1H), 2.35 (m, 2H), 2.20 (m, 2H), 2.10 (s, 1H), 1.61 (m, 2H). ^13^C NMR (126 MHz, MeOD) δ 174.5, 84.6, 69.8, 68.2, 43.11, 37.0, 15.6. HR-ESI (m/z): [M + H]^+^ calcd for C_7_H_10_O_3_ 165.0528, found 165.0520.

**2.3 Solid-phase Peptide synthesis**

**2.3.1 General Information**

Peptide standards such as ACAT1 (IHMGS-Cys(cr)-AENTAK) and HSD17B10 (V-Cys(cr)-NFLASQVPFPSR) were synthesized by a solid-phase peptide synthesis (SPPS) strategy that combined automated and manual steps. For each peptide, the C-terminal portion of the sequence (e.g., AENTAK in ACAT1) was first assembled using automated microwave-assisted coupling and deprotection cycles as described below. Upon completion of the initial segment, the peptide-bound resin was removed from the synthesizer and subjected to manual coupling of Fmoc-Cys(STmp)-OH under standard HATU-mediated conditions. Following cysteine incorporation, the Tmp protecting group on the side chain thiol was selectively removed by a mixture of DTT and NMM. A crotonation modification was then introduced via Michael addition by treating the side-chain deprotected cysteine with (*E*)-tert-butyl but-2-enoate in the presence of DIPEA. After the crotonation step, the modified resin was thoroughly washed, dried, and returned into the peptide synthesizer for completion of the remaining N-terminal sequence.

**2.3.2 Automated solid-phase peptide synthesis**

Automated Fmoc-based solid-phase peptide synthesis was performed on a CEM Liberty Blue 2.0 synthesizer at 0.1 mmol scale using DMF as solvent. The resin was pre-swelled in DMF. Each cycle involved Fmoc deprotection with 10% piperidine in DMF (4 mL) at 90 °C for 1 min, followed by DMF washes (4 × 4 mL). Coupling was carried out with 5.0 equiv Fmoc-amino acid, 10.0 equiv DIC, and 5.0 equiv Oxyma under microwave heating at 90 °C for 2 min. The process was repeated for each residue in the sequence.

**2.3.3 Manual Coupling of Cys(S-Tmp) and deprotection of Tmp group**

Resin (0.1 mmol) was pre-swelled in DMF. A coupling solution containing HATU (1.8 equiv), Fmoc-Cys(S-Tmp)-OH (2.0 equiv), and DIPEA (4.0 equiv) in 5 mL DMF was freshly prepared and added to the resin. The mixture was shaken at room temperature for 1 hour, then washed with DMF (6 × 5 mL). A second identical coupling was performed overnight, followed by the same washing and vacuum drying. For Tmp deprotection, DTT (16 equiv) and NMM (5 equiv) were dissolved in 5 mL DMF and added to the resin. After shaking for 30 min, the solution was removed and the resin washed (6× 5 mL DMF). The deprotection step was repeated once more under the same conditions. The resin was washed again and used directly for the next step.

**2.3.4 Michael Addition of Crotonate to Cysteine**

(*E*)-tert-butyl but-2-enoate (5.0 equiv) and DIPEA (10.0 equiv) were dissolved in 5 mL DMF and added to the resin containing deprotected cysteine. The mixture was shaken at room temperature for 1 hour, washed with DMF (6× 5 mL), then subjected to a second identical reaction overnight. Afterward, the resin was washed with DMF, DCM (3× 5 mL), and methanol (3× 5 mL), then dried and stored at -20 °C for future use.

**2.3.5 Acid Cleavage Procedure**

Each peptide was cleaved from resin using a freshly prepared cocktail of TFA/TIS/H₂O/DODt (92.5:2.5:2.5:2.5, v/v), with 10 mL prepared per cleavage. Approximately 5 mL was added to the peptide-bound resin and shaken vigorously at room temperature for 2.5 hours. The mixture was collected, and the resin was rinsed with an additional 3 mL of the same cocktail. The combined flow-through (FT) was partially evaporated by airflow, then diluted to 50 mL with cold (-20 °C) diethyl ether. After vortexing and centrifugation (20,000 g, 15 min, 4 °C), the peptide pellet was washed once more with cold ether and centrifuged again. The resulting pellet was air-dried under a KimWipe in a fume hood for ~2 hours and stored at -20 °C. For PRM analysis, 1 mg of crude peptide was dissolved in 1 mL of 0.1% formic acid; 1 µL was spiked into the sample for co-elution, and 1 µL was dried as a control.

**2.3.6 Pre-test to confirm the Ccr linkage**

Truncated synthetic peptide CcrNFLA from HSD17B10 was synthesized by SPPS above. To confirm its thioether linkage, ^1^H and ^13^C NMR spectra were tested. ^1^H NMR (500 MHz, DMSO-*d*_6_) δ 8.70 (s, 1H), 8.21 – 8.12 (m, 1H), 8.03 (d, *J* = 8.4 Hz, 1H), 7.96 (s, 1H), 7.49 (s, 1H), 7.24 (s, 4H), 7.19 (s, 1H), 7.00 (s, 1H), 4.60 (s, 1H), 4.45 (s, 1H), 4.38 – 4.26 (m, 1H), 4.28 – 4.13 (m, 1H), 3.82 (m, 1H), 3.23 – 3.17 (m, 2H), 3.08 (s, 1H), 2.96 – 2.87 (m, 1H), 2.82 (d, *J* = 12.1 Hz, 1H), 2.80 – 2.75 (m, 1H), 2.66 – 2.60 (m, 2H), 2.40 (m, 2H), 1.62 (s, 1H), 1.66 – 1.60 (m, 1H), 1.55 – 1.45 (m, 2H), 1.29 (m, 6H), 0.88 (m, 6H). ^13^C NMR (126 MHz, DMSO-*d*_6_) δ 174.4, 173.0, 172.9, 172.0, 171.8, 170.9, 170.8, 138.2, 138.2, 129.6, 129.6, 128.5, 126.7, 55.4, 54.6, 51.3, 50.2, 47.9, 41.7, 41.0, 40.5, 36.5, 36.0, 24.5, 23.6, 22.0, 21.6, 21.5, 17.5. MALDI-TOF (m/z): [M+H]^+^ and [M+Na]^+^ found at 653.5 and 675.4, respectively.

Before proceeding with further studies, we performed a preliminary pre-cleavage experiment to confirm the chemical nature of the Ccr linkage. Unmodified peptide VCNFLASQVPFPSR and IHMGSCAENTAK were first cleaved from the resin, purified by HPLC, and its identity confirmed by MALDI-MS. The purified peptide was then incubated with (*E*)-tert-butyl but-2-enoate (5.0 equiv) and DIPEA (5.0 equiv) in PBS (0.5 mL) for 1 hour. Subsequent MS/MS analysis revealed formation of the Michael addition product with the tert-butyl ester group intact. These results demonstrate that the modification proceeds via Michael addition rather than thioester formation, confirming the chemical nature of the Ccr linkage.


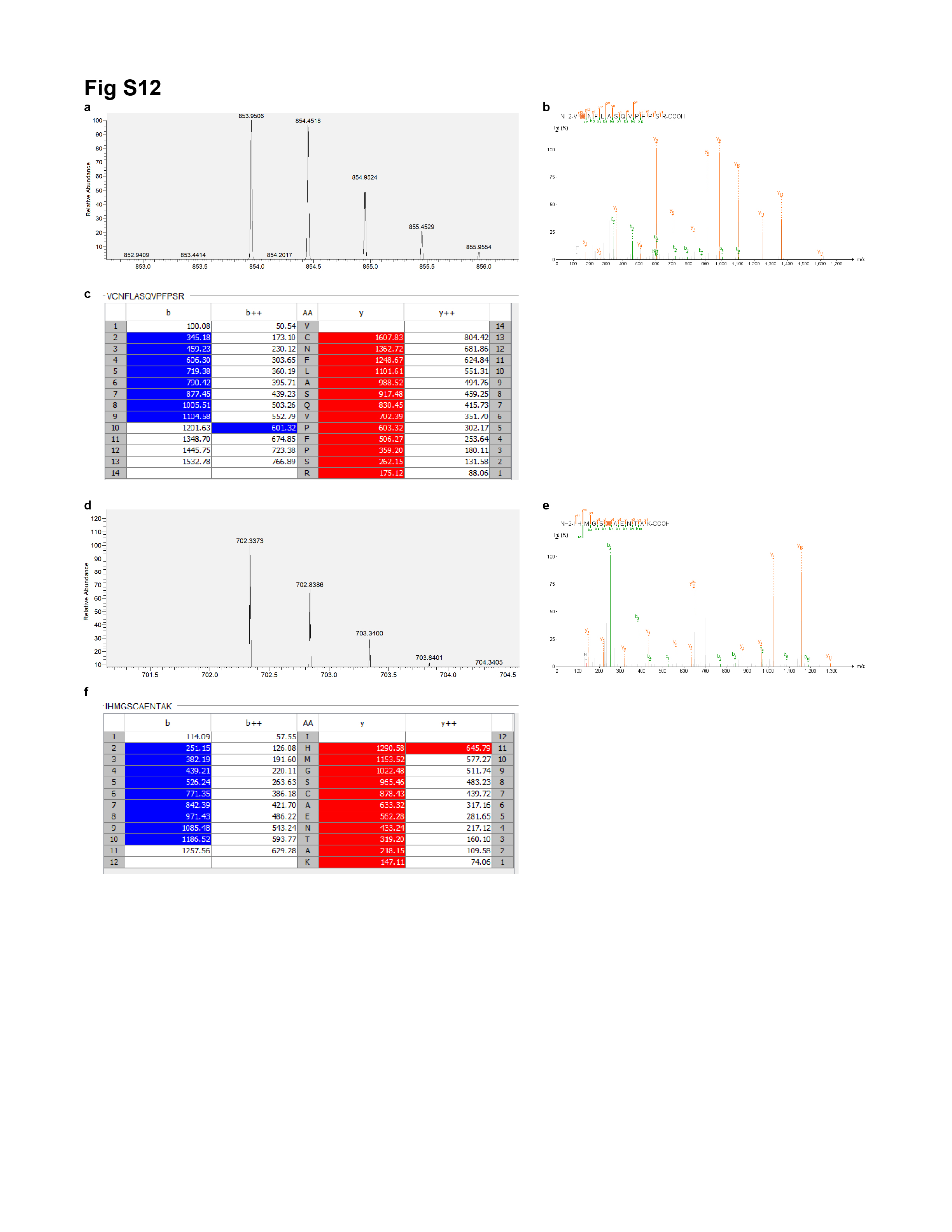


**Figure S12**. (a–c) VCNFLASQVPFPSR peptide conjugation with (*E*)-tert-butyl but-2-enoate (crotonyl-OtBu): (a) The doubly charged molecular ion was observed in the MS1 spectrum at m/z 853.9508, corresponding to [M + 2H]^2+^. (b) MS/MS spectrum showed crotonyl-OtBu formed in Cys, and (c) fragment-ion assignment. (d–f) IHMGSCAENTAK after conjugation with crotonyl-OtBu: (d) The doubly charged molecular ion was observed in the MS1 spectrum at m/z 702.3373, corresponding to [M + 2H]^2+^, (e) MS/MS spectrum showed crotonyl-OtBu formed in Cys, and (f) fragment-ion assignment.

1. **Biological experiments materials and methods**

**3.1 Cell culture**

LNCaP (CRL-1740), 22Rv1 (CRL-2505) and HEK293T (CRL-3216) were obtained from the ATCC. Hepa1-6 and NIH3T3 were kind gifts from Dr. Emmanuel Thomas and Dr. Zhipeng Meng, respectively. LNCaP and 22Rv1 were cultured in RPMI-1640, Hepa1-1, NIH3T3 and HEK293T cells were cultured in DMEM (Gibco, Invitrogen), supplemented with 10% fetal bovine serum (FBS) and 100 units/ml penicillin/streptomycin at 37°C in a humidified incubator under 5% CO_2_. All cell culture reagents were obtained from ThermoFisher, except for sera, FBS, which were obtained from GeminiBio. For transient transfection, cells were grown on cell culture dishes or plates to 70% confluence and transfected with indicated plasmids using PolyJet (SignaGen Laboratories) at a 3:1 ratio of transfection reagent/DNA in Opti-MEM media (ThermoFisher) for 18 hours. To create knockout cell lines, LNCaP cells were infected with lentivirus containing single guide RNAs (sgRNAs) targeting BDH1 (CATAAGTCCGACGGCCAATC) and ECHS1 (CGCCAGGCGGGACAGCGAAC). The sgRNAs were synthesized by GenScript Biotech. Cells were selected with 2.5 mg/mL puromycin, and the resulting CRISPR knockout pools were used in the study.

**3.2 Western blotting**

The cells were lysed in RIPA buffer (ThermoFisher Scientific, cat no: 89901) and Protein concentrations were measured using Pierce BCA Protein Assay kit (Thermo Fisher Scientific, 23225). For reducing SDS-PAGE, lysates were diluted 1:1 with 2X Laemmli sample buffer, heated at 95°C for 5 min before resolution on 12% SDS-PAGE gels. Nonreducing SDS-PAGE was performed in the same manner, except that β-mecaptoethanol was omitted from sample buffer. Blots were probed with antibodies against the following proteins: PRDX3 antibody (1:2500, Santa Cruz Biotechnology, sc-59663), GAPDH (1:5000, Santa Cruz Biotechnology, sc-47724) and β-actin antibody (1:5000, Proteintech, 20536-1-AP). Following incubation with the appropriate secondary horseradish peroxidase–conjugated antibodies, blots were developed using Pierce™ ECL Western Blotting Substrate (ThermoFisher Scientific, 32106). The protein marker used in this study was Biorad precision Plus Dual Color standards. The western blot images were taken using Bio-Rad ChemiDoc™ MP Imaging System. Densitometry of images was carried out via the ImageJ Analyze Gels (NIH) module and data analysis and graphing were performed in GraphPad Prism (v.10).

**3.3 Colony formation assay**

LNCaP cells were seeded at 5,000 cells per well in 6-well plates and treated the following day with sodium acetoacetate (Acac; Biosynth, Cat no: FA166793) or Acac-alkyne or hydroxybutyric acid sodium salt (ChemCruz, Cat no: sc-229050A) at 5 mM or 10 mM concentration for 3 days. Then the cells were cultured in fresh RPMI. After 14 days, cells were fixed with 1% paraformaldehyde and stained with 0.05% crystal violet for 30 minutes. Following three washes with ddH₂O, images were captured, and colony numbers were quantified using ImageJ software.

**3.4 Metabolic labeling in mammalian cells with Acac-alkyne probe**

The bioorthogonal probe (Acac-alkyne) was dissolved in PBS to prepare a 1 M stock solution. For metabolic labeling of cellular proteins, LNCaP cells were incubated with 10 mM Acac-alkyne in RPMI for 2 hours at 37 °C in a humidified incubator. For Acac and Acac-alkyne competition assay, LNCaP cells were co-treated with sodium acetoacetate at the indicated concentrations and 5 mM Acac-alkyne for 2 hours. Labeled cells were then washed with cold PBS, harvested, flash-frozen in liquid nitrogen, and stored at - 80 °C.

**3.5 CuAAC click reaction and in-gel fluorescence analysis**

To detect Acac-alkyne-labeled proteins, a copper(I)-catalyzed azide-alkyne cycloaddition (CuAAC) reaction was performed. Briefly, 150 µg of cell lysate in 0.1% Triton/PBS was incubated with 100 µM azido-rhodamine, 1 mM freshly prepared tris(2-carboxyethyl)phosphine hydrochloride (TCEP), 0.1 mM tris[(1-benzyl-1H-1,2,3-triazol-4-yl)methyl]amine (TBTA), and 1 mM CuSO₄ for 1 hour at 37 °C in the dark with gentle agitation. Following the click reaction, 4× Laemmli sample buffer was added, and the samples were boiled for 10 minutes at 95 °C before loading onto a 10% SDS-PAGE gel for separation. Labeled proteins were visualized using a ChemiDoc MP Imaging System (Bio-Rad) with a rhodamine filter. After in-gel fluorescence imaging, the gel was stained with Coomassie Brilliant Blue for total protein visualization.

**3.6 ROS measurements**

The ROS assay was carried out via staining with 5 μM 5- (and -6)-chloromethyl-2′,7′-dichlorofluorescein diacetate (CM-H2DCFDA; ThermoFisher Scientific, C6827). Briefly, cells at equivalent confluency were treated with 10 mM Acetoacetate (Ambeed, A1155657) for 24 hours and collected through trypsinization, washed in ice-cold Ca^2+^- and Mg^2+^-free 1X Hank’s balanced salt solution (HBSS, Life Technologies, 14175-095), and incubated with freshly prepared CM-H_2_DCFDA for 15 min at 37 °C. The cells were then washed and resuspended in 1× HBSS before detection of fluorescent signal. The x axis represents FITC channel (FL1) fluorescence intensity in log-scale and the y axis represents the number of cells. ROS assay flow cytometric profiles were generated and analyzed on BD FACSAria™ Fusion Flow Cytometer and FlowJo v10. Data analysis and graphing were performed in GraphPad Prism (v.10).

**3.7 Plasmids and cloning**

Full-length PRDX3 were cloned into the pCMV-FLAG vector (GenScript Biotech). The primer sequences for to generate PRDX3 C229A, C229S, C229E mutants were used in this study are listed below. The Q5^®^ Site-Directed Mutagenesis Kit (NEB) were used for cloning.

| Primer name | Sequence (5' to 3') |
| --- | --- |
| Prdx3_C229A_F | catggagaagtcgccccagcgaactggacaccggattctcctac |
| Prdx3_C229A_R | tgtccagttcgctggggcgacttctccatgtgtttctacatactgg |
| Prdx3_C229S_F | catggagaagtctccccagcgaactggacaccggattctcctac |
| Prdx3_C229S_R | tgtccagttcgctggggagacttctccatgtgtttctacatactgg |
| Prdx3_C229E_F | catggagaagtcgaaccagcgaactggacaccggattctcctac |
| Prdx3_C229E_R | tgtccagttcgctggttcgacttctccatgtgtttctacatactgg |

**3.8 Cellular thermal shift assay**

LNCaP cells were incubated with 5 mM Acetoacetate for 24 hours and then harvested in PBS. The cells were lysed in RIPA buffer (ThermoFisher Scientific, cat no: 89901) and 40 μl of treated and non-treated cell lysates were transferred into PCR tubes, heated at 47, 57, 60, 63, 67, 70, 77°C for 3 min in PCR Thermocycler. The lysates then were transferred to a new Eppendorf tube and followed by centrifugation at 17,000 g for 10 min. 10 µg of each supernatant was subjected to immunoblotting analysis using PRDX3 antibody (Santa Cruz Biotechnology, sc-59663). The Western Blot images were taken using Bio-Rad ChemiDoc™ MP Imaging System. Densitometry of images was carried out via the ImageJ Analyze Gels (NIH) module. Data analysis and graphing were performed in GraphPad Prism (v.10).

1. **Chemical proteomics study**

**4.1** **Chemical proteomics of Acac-alkyne, Cro-alkyne and Bhb-alkyne** **labeled proteins**

LNCaP cells were treated with 10 mM Acac-alkyne for 24 hours. Cells were harvested, lysed and centrifuged at 20,000g for 10 min to remove cellular debris. Protein concentrations were determined by the BCA assay (Pierce). The cell lysates (2 mg protein) were then clicked with acid cleavable azido-DADPS-biotin in the presence of TCEP, TBTA, and CuSO_4_ as described above. Methanol precipitated and washed protein pellets were again resuspended in 4% SDS buffer. Protein concentrations were determined, and equal amounts of each protein sample were diluted by volume with 50 mM triethanolamine buffer to 1% SDS (~1.6 mg/mL protein). Then pre-washed streptavidin agarose beads (~50 μL slurry; ThermoFisher) were added to each sample. The protein and beads mixtures were incubated at room temperature on a rotator for 4 h. The beads were then washed 3 times with PBS and then 3 times with ddH_2_O and transferred into spin-columns (ThermoFisher). The beads were then added with 6 M urea and then incubated with 10 mM dithiothreitol for 0.5 h, followed by treatment with 20 mM iodoacetamide for another 0.5 h in the dark. After that, the beads were washed with PBS and digested with 2 μg of trypsin in PBS buffer at 37 °C overnight. The beads were again washed 6 times with PBS and ddH_2_O. Finally, the beads were resuspended with 2 % formic acid in ddH_2_O (200 μL) to cleave the DADPS linker. The elution was repeated twice and the supernatants were pooled and dried by SpeedVac for LC-MS/MS analysis. For Cro-alkyne and Bhb-alkyne, we used the same procedures as acac-alkyne.

**4.2 Quantitative chemical proteomics of Acac-alkyne labeled proteins**

For the quantitative identification enrichment proteins of Acac-alkyne, LNCaP cells were grown to 80% confluence in 15 cm dish with 10 mM Acac-alkyne or 10 mM Acac for 24 h. The cells were collected and lysed in 1 mL ice-cold PBS buffer containing EDTA-free Pierce HaltTM protease inhibitor cocktail with sonication. The cell lysates were collected by centrifugation (20,000 g, 15 min) at 4 ^o^C to remove the debris. The protein concentration was determined by using the BCA protein assay kit. 1 mL cell lysates (2 mg/mL) were reacted with 1 mM CuSO_4_, 100 μM TBTA ligand, 100 µM azide-biotin, and 1 mM TCEP for 1 h at room temperature. The resulting click-labeled lysates were centrifuged at 18000 g for 10 min at 4 ^°^C and washed twice with 1 mL cold methanol. The proteins were resuspended in 1 mL PBS containing 1.2% SDS. 100 µL streptavidin beads (Thermo Fisher Scientific) were washed three times with 1 mL PBS, and resuspended in 5 mL PBS, which was added to the protein solution. The resulting solution was incubated for 4 h at 29 ^°^C, followed by washing with 5 mL PBS for three times, and 5 mL ddH_2_O for three times. The resulting beads were resuspended in 500 µl PBS containing 6 M urea and 10 mM DTT and incubated at 37 ^°^C for 30 min, followed by addition of 20 mM iodoacetamide for 30 min at 37 °C in the dark. The beads were then collected by centrifugation and resuspended in 200 µL PBS containing 2 M urea, 2 µg trypsin. Trypsin digestion was performed at 37 ^°^C with rotation overnight and the beads were washed with 200 µl ddH_2_O for three times. For dimethyl labeling, per 100 μL peptides were reacted with 4 μL of 4% “light” formaldehyde and “heavy” formaldehyde (CD_2_O), respectively. The resulting solution was treated with 4 μL 0.6M sodium cyanoborohydride and incubated at RT for 1 h. The reaction was quenched by adding 16 μL 1% ammonia and 8 μL 5% formic acid. The “light” and “heavy” samples were combined and subjected for fractionation. Peptides were desalted using C18 solid-phase extraction cartridges, separated into 3 fractions and dried under vacuum.

**4.3 LC-MS/MS analysis**

LC-MS/MS was performed on an Exploris 240 Orbitrap mass spectrometer (Thermo Fisher Scientific) coupled with a Vanquish Neo UHPLC. Mobile phase A was 0.1% formic acid in H_2_O, and mobile phase B was 0.1% formic acid, 80% acetonitrile in H_2_O. Flow rate was 10 μL/min for loading and 0.3 μL/min for eluting. Under the positive-ion mode, full-scan mass spectra were acquired over the m/z range from 350 to 1600 using the Orbitrap mass analyzer with mass resolution of 120,000. MS/MS fragmentation was performed in a data dependent mode, of which the most intense ions in a 3-second cycle was selected for MS/MS analysis a resolution of 30,000 using collision mode of HCD. Other important mass parameters: isolation window, 1.6 m/z units; default charge, 2+; normalized collision energy, 26%; dynamic exclusion, 45.0 s.

**4.4 Mitochondrial isolation and sample preparation for proteomics analysis**

Mitochondria were isolated using differential centrifugation. Briefly, cells were harvested and washed twice with ice-cold phosphate-buffered saline (PBS), then resuspended in mitochondrial isolation buffer (250 mM sucrose, 1 mM EDTA, 10 mM HEPES, pH 7.4) supplemented with protease and phosphatase inhibitors. The cell suspension was homogenized on ice using a glass Dounce homogenizer with ~30 strokes to ensure efficient cell lysis without damaging mitochondria. The homogenate was centrifuged at 800 × g for 10 minutes at 4 °C to remove nuclei and debris. The supernatant was collected and further centrifuged at 10,000g for 15 minutes at 4 °C to pellet the crude mitochondrial fraction. The mitochondrial pellet was washed once with isolation buffer and resuspended in appropriate lysis buffer (8 M urea, 50 mM Tris-HCl, pH 8.0) for downstream proteomics assays.

The protein concentration was determined using a BCA assay. For digestion, proteins were reduced with 10 mM dithiothreitol at 37 °C for 1 hour, followed by alkylation with 20 mM iodoacetamide in the dark at room temperature for 30 minutes. The urea concentration was then diluted to less than 2 M with 50 mM Tris-HCl (pH 8.0), and sequencing-grade trypsin (Promega) was added at a 1:50 (enzyme:substrate) ratio for overnight digestion at 37 °C. Peptides were desalted using C18 solid-phase extraction cartridges and dried under vacuum. For high-pH reversed-phase fractionation, peptides were reconstituted in 10 mM ammonium formate (pH 10.0) and fractionated using stepwise elution with increasing concentrations of acetonitrile. Each fraction was dried and stored at −80 °C until LC-MS/MS analysis.

**4.5 Data analysis**

Search of probe modified sites was performed by using FragPipe GUI v18.0 with MSFragger (version 3.3), Philosopher (version 4.0.0), and IonQuant (version 1.7.5).^1-3^ Precursor mass tolerance was set -20 to 20 ppm. Fragment mass tolerance was set as 20 ppm. Enzyme specificity was set to trypsin with up to 2 missed cleavages. Peptide length was set 7 to 50, and peptide mass range was set 500 to 5000 Da. Peptides were required to achieve a peptide false-positive rate below 1%. Raw files were searched against the Homo sapiens UniProt reference proteome (UP000005640) and a reversed/decoy database was used for false discovery rate estimation. Cysteine carboxyamidomethylation (+57.02146 Da), methionine oxidation (+15.9949 Da), and protein N-terminal acetylation (+42.0106 Da) were set as variable modification. Cysteine modification (+267.1577 Da and +427.1867 Da) was set as variable modification. The max variable modification on a peptide was set as 3, max combinations 5000. For quantitative analysis, dimethyl labeling was specified as light (+28.0313 Da) and heavy (+32.06312 Da) modifications on lysine residues and peptide N-termini. Quantification was performed at the peptide level using ion intensity. For enrichment analysis, protein enrichment ratios were calculated as Log₂(L/H). Proteins with Log₂ (L/H) enrichment ≥ 1 were considered significantly enriched.

The mass spectrometry proteomics data have been deposited to the ProteomeXchange Consortium (http://proteomecentral.proteomexchange.org) via the iProX^4-5^ partner repository with the dataset identifier PXD066371.

1. **Supplementary tables**

**Table S1**. Site-specific identification of cysteine crotonation and another cysteine modifications (Cys+427) discovered by the Acac-alkyne probe.

1. **Characterization of the compounds**


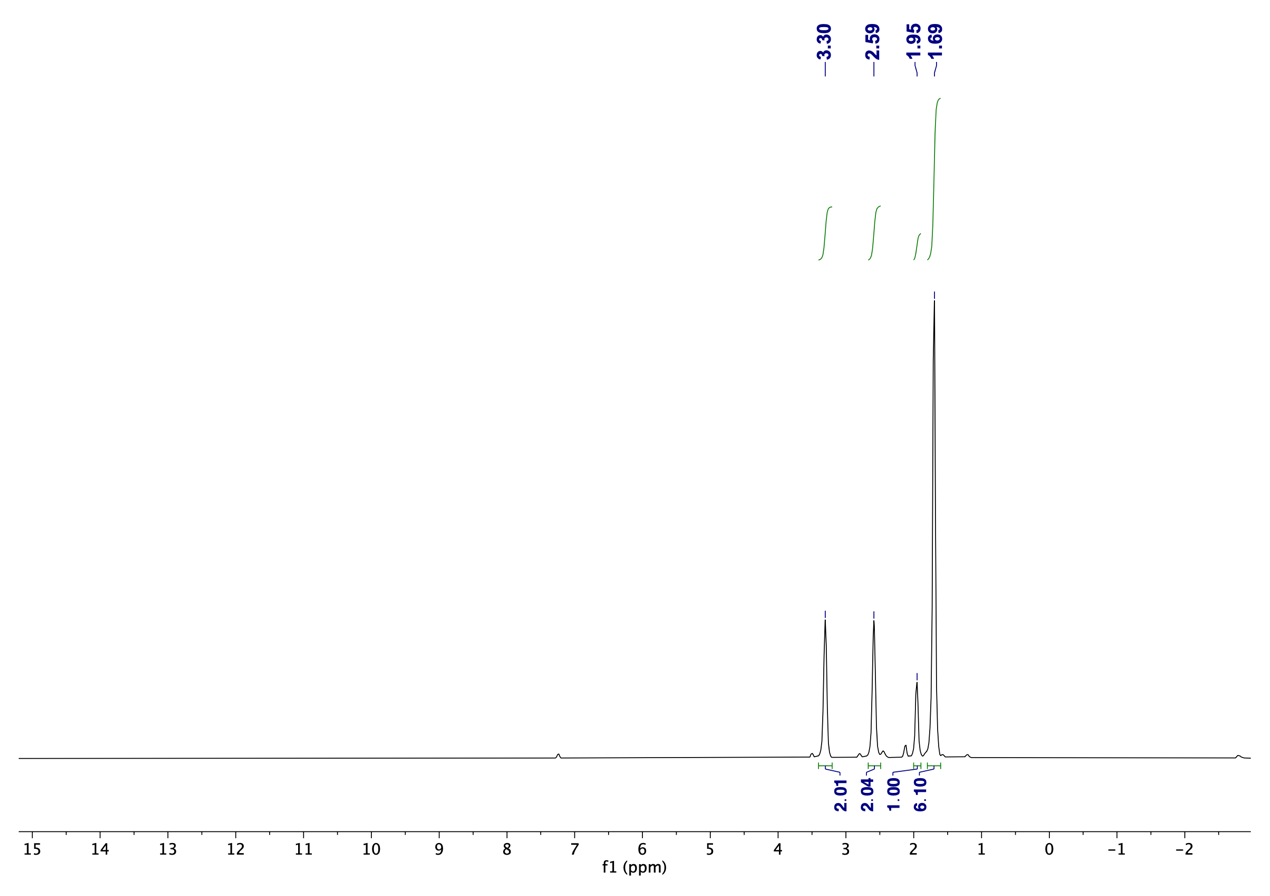


^1^H NMR (500 MHz) spectrum of compound **2** in CDCl_3_.


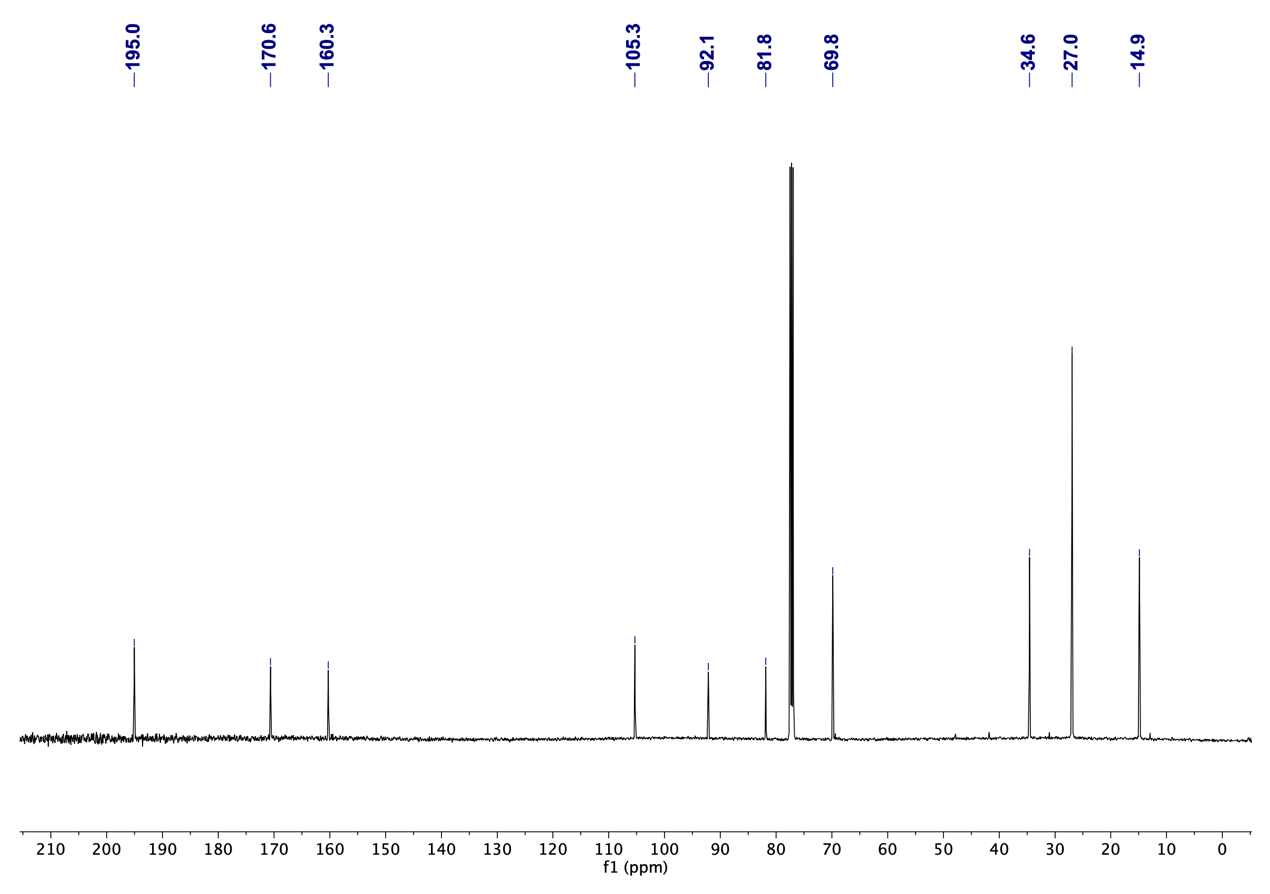


^13^C NMR (126 MHz) spectrum of compound **2** in CDCl_3_.


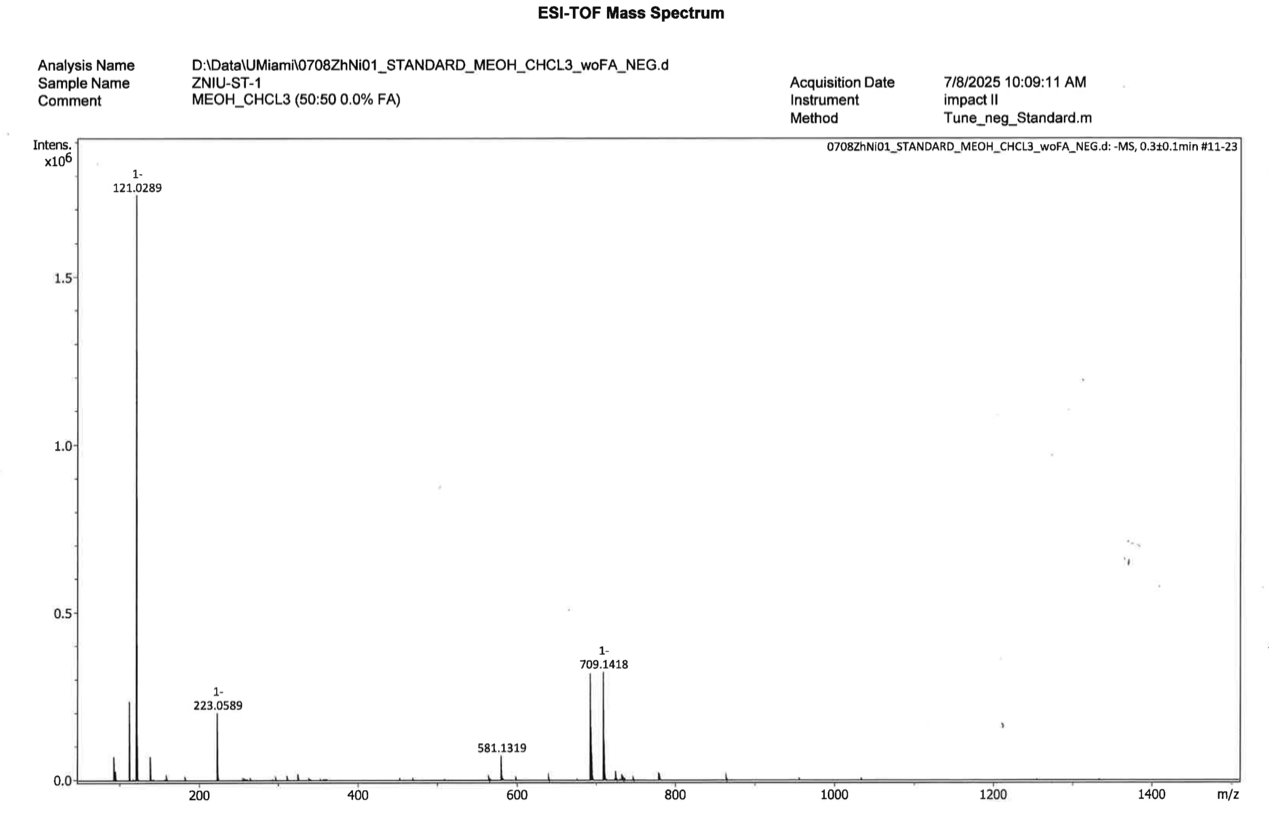


HR-MS spectrum of compound **2.**


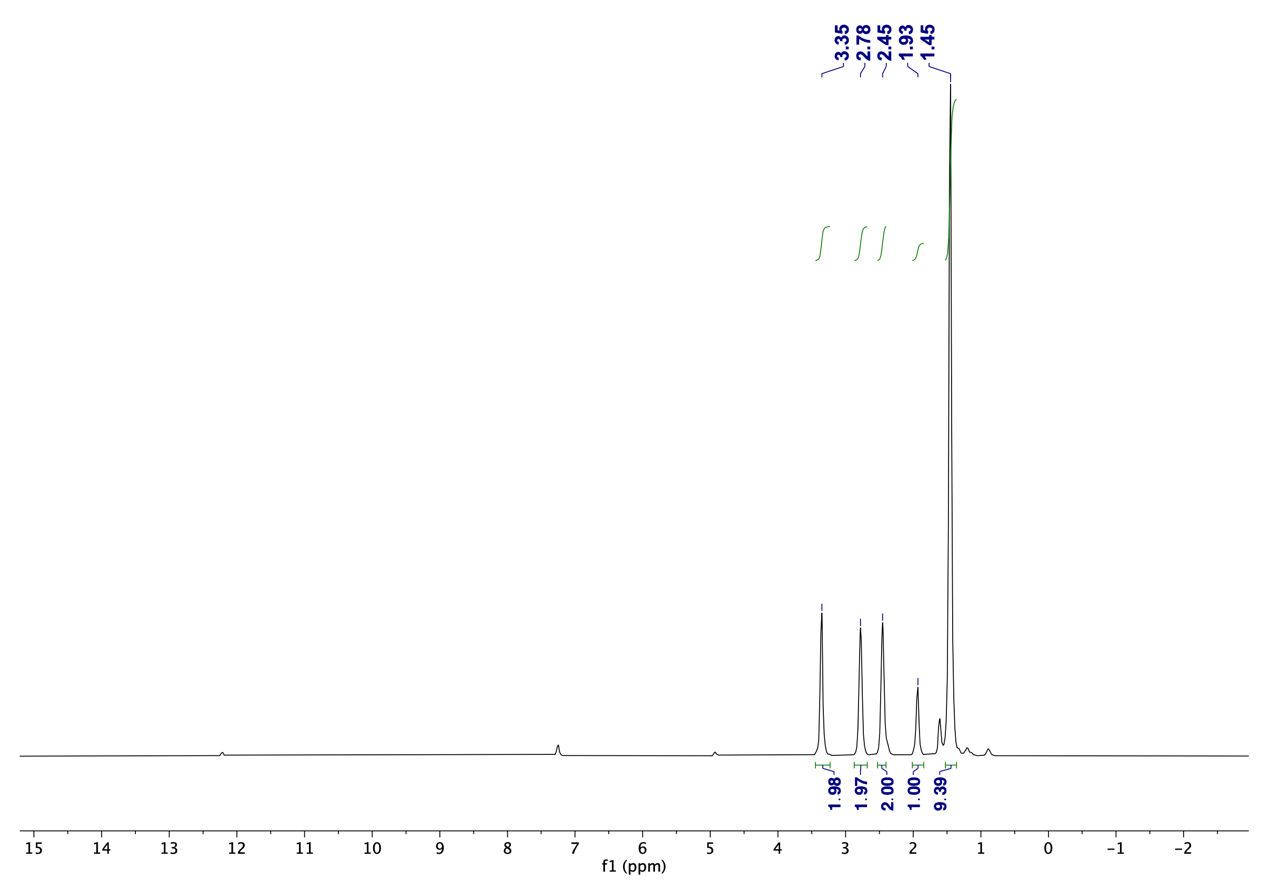


^1^H NMR (500 MHz) spectrum of compound **3** in CDCl_3_.


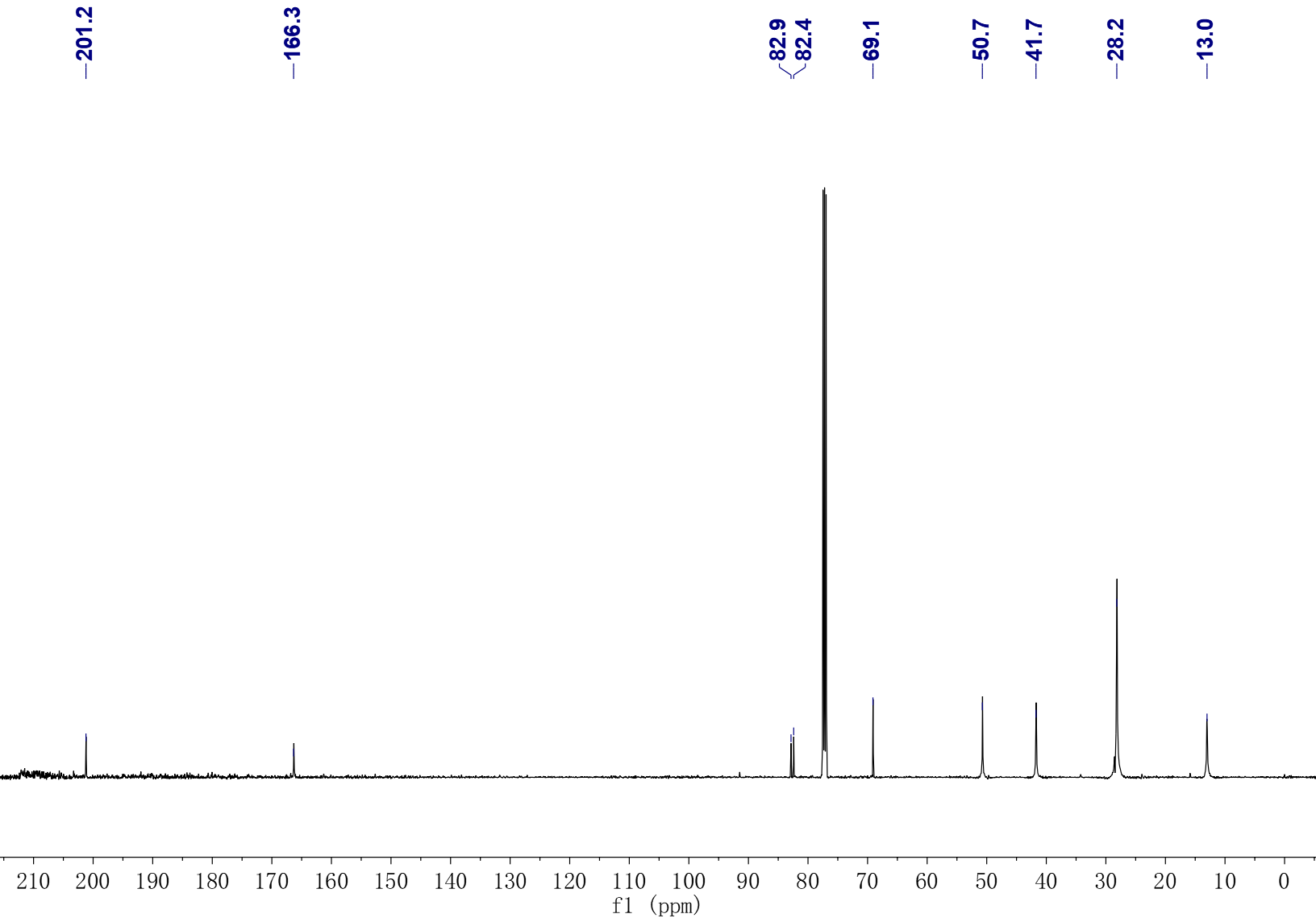


^13^C NMR (126 MHz) spectrum of compound **3** in CDCl_3_.


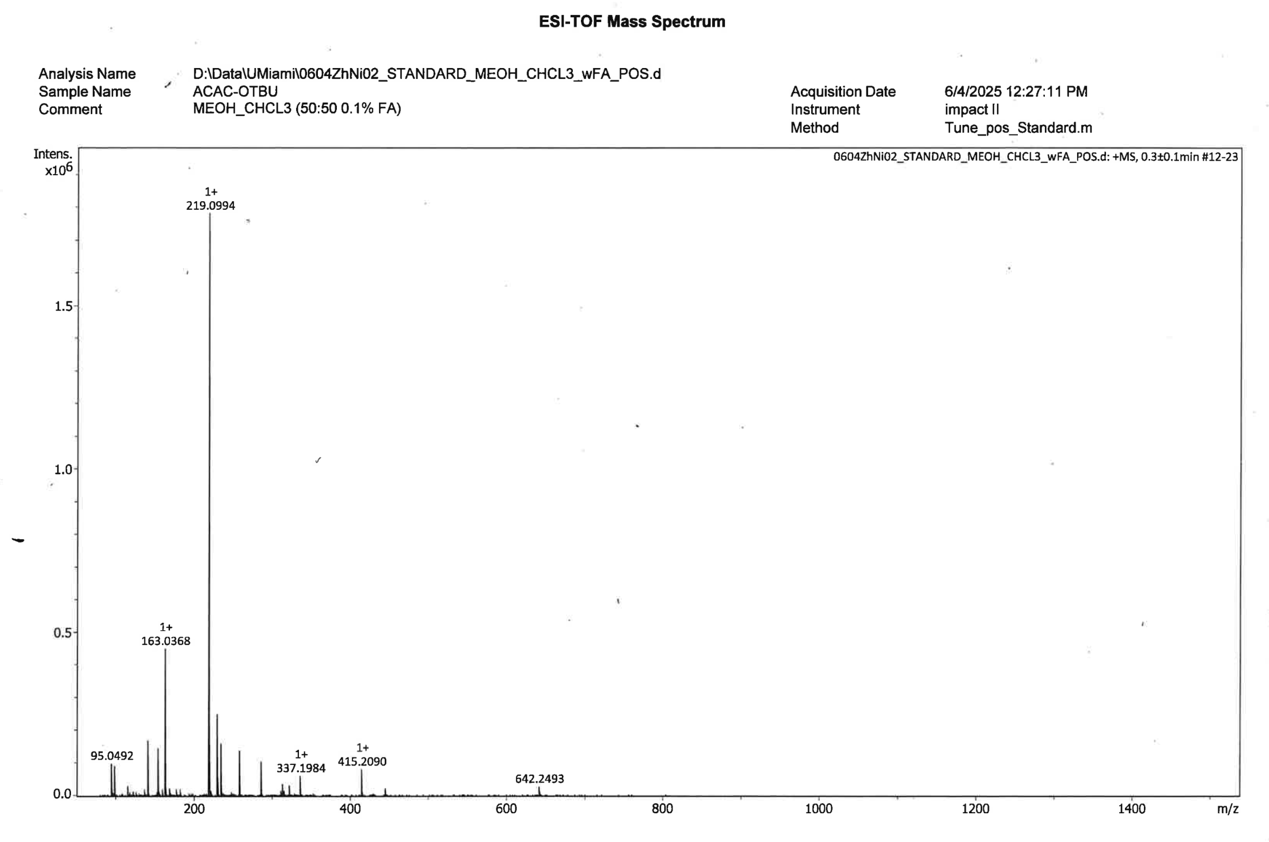


HR-MS spectrum of compound **3**.


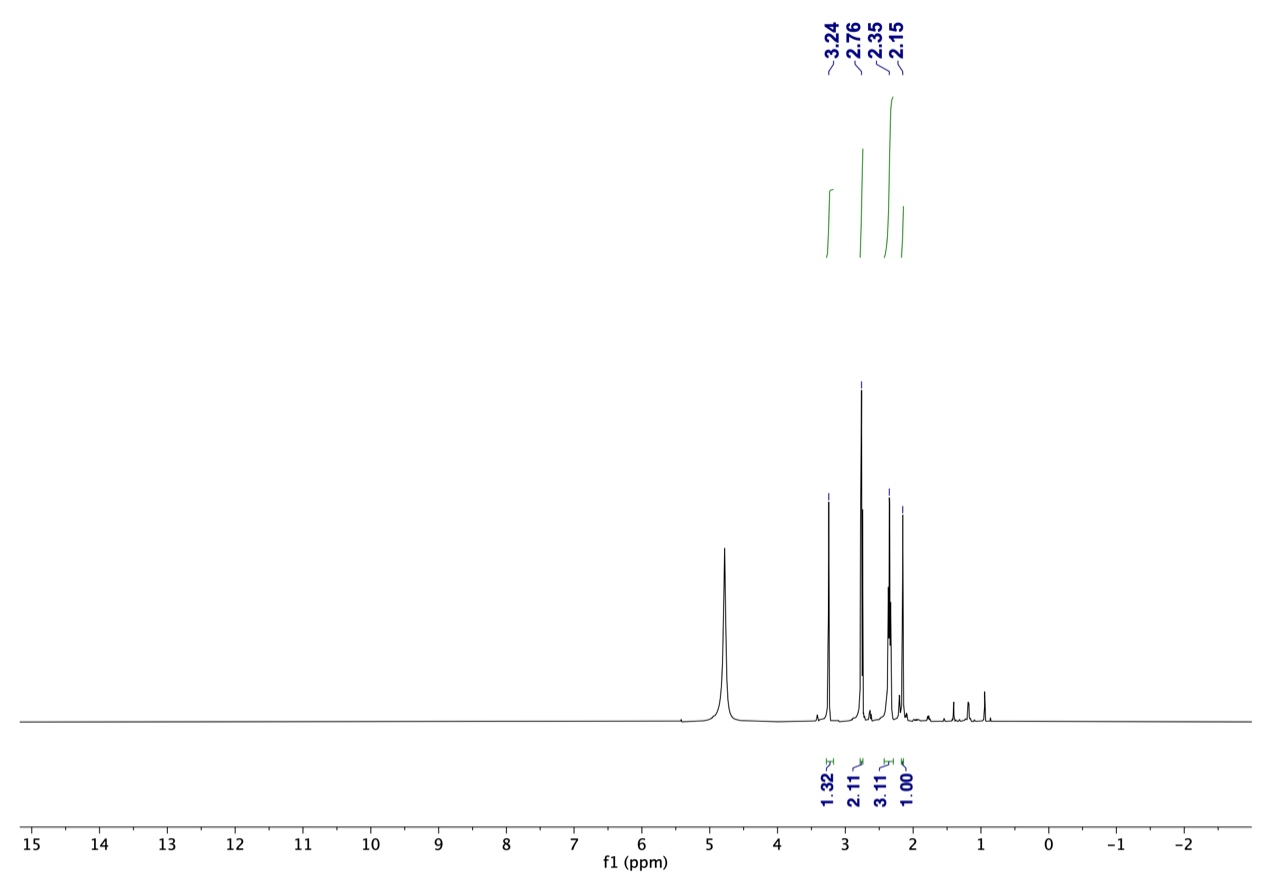


^1^H NMR (500 MHz) spectrum of compound **4** in CD_3_OD.


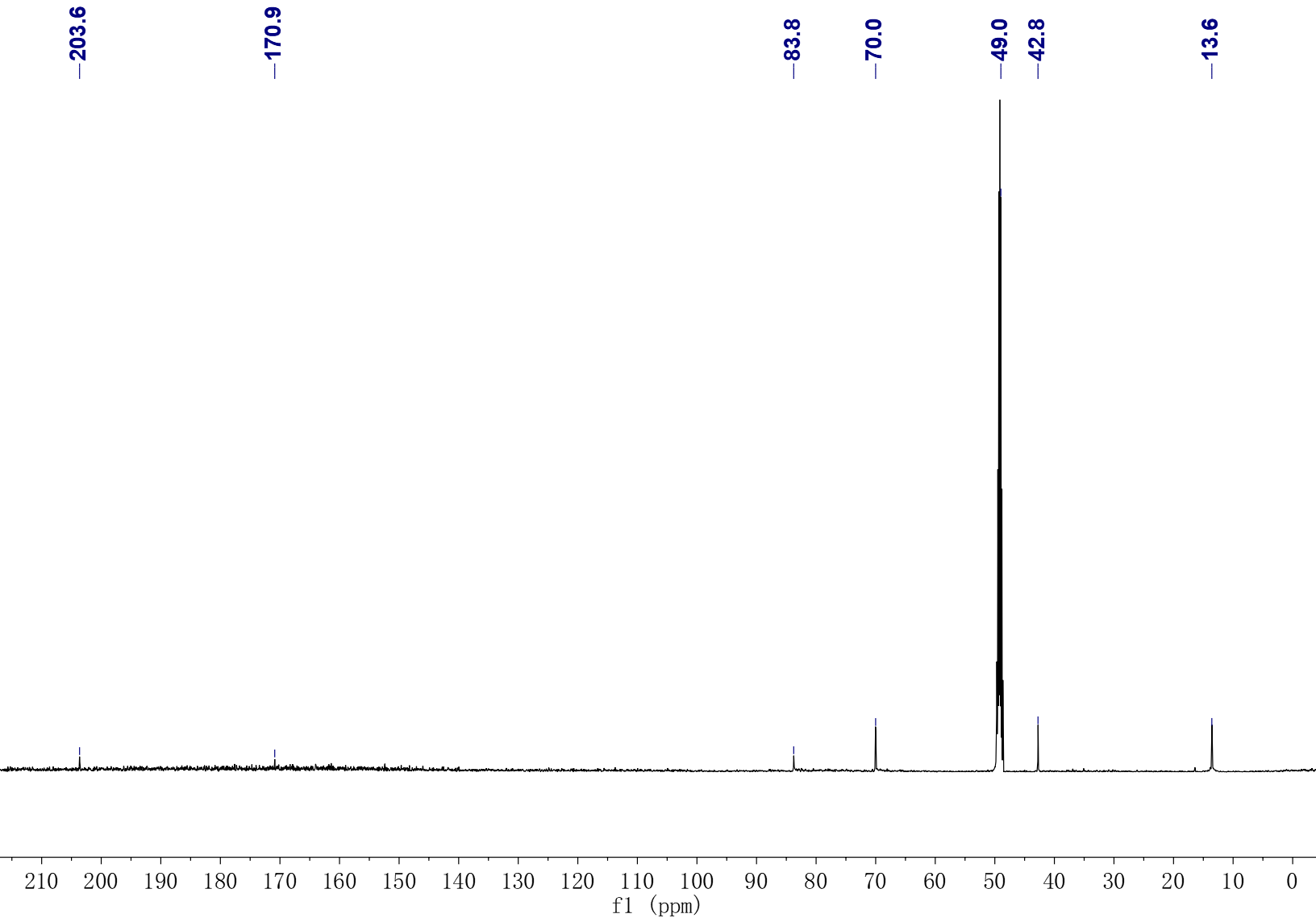


^1^H NMR (126 MHz) spectrum of compound **4** in CD_3_OD.


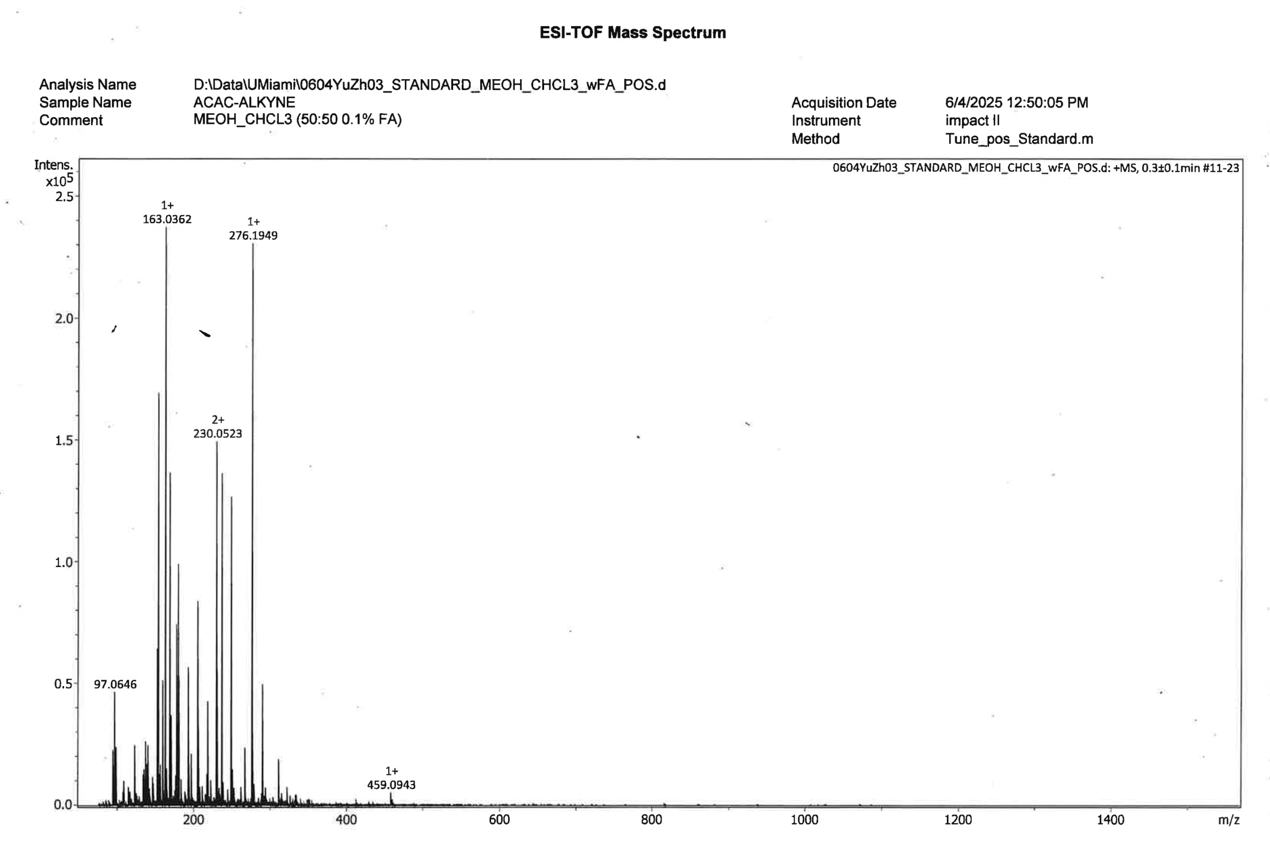


HR-MS spectrum of compound **4**.


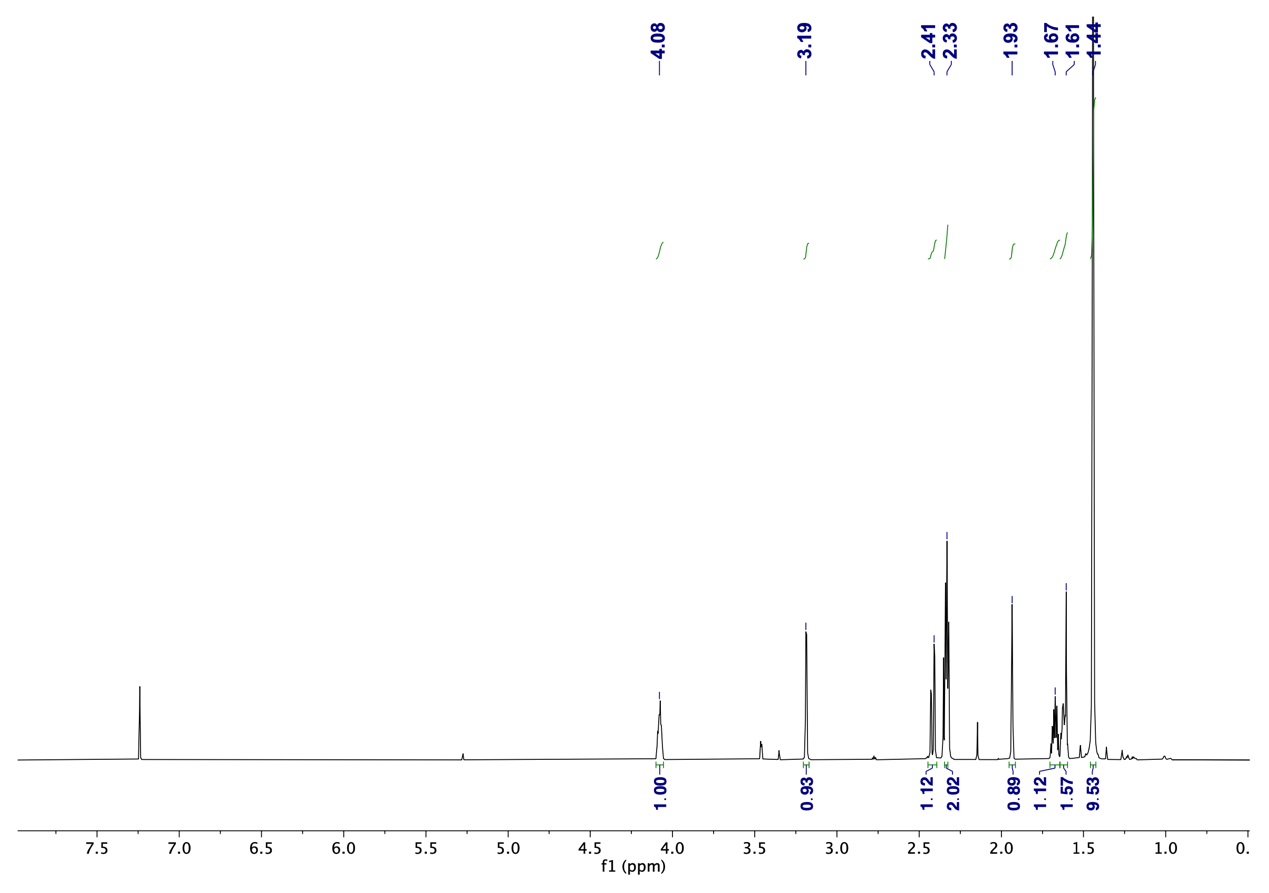


^1^H NMR (800 MHz) spectrum of compound **5** in CDCl_3_.


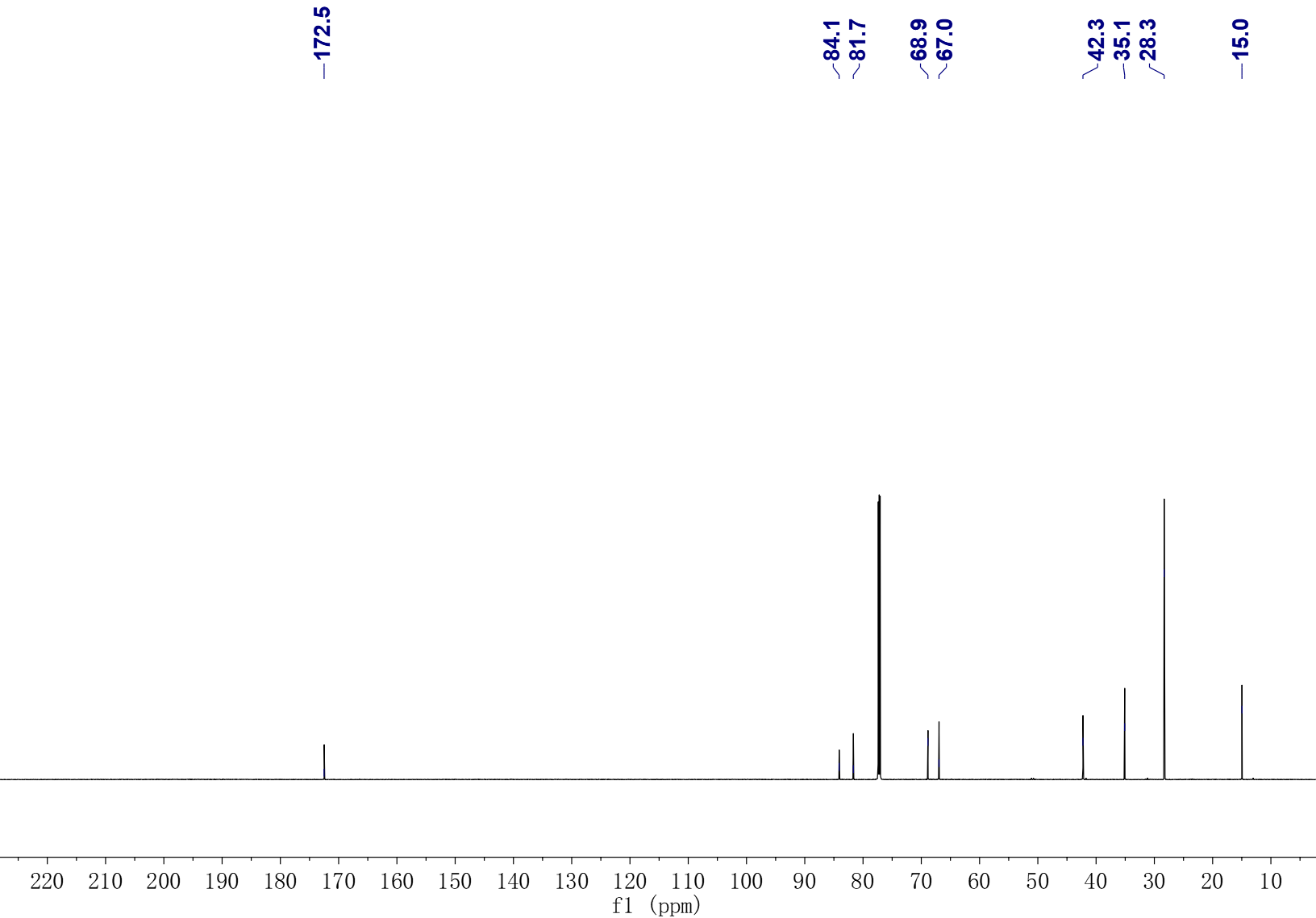


^13^C NMR (201 MHz) spectrum of compound **5** in CDCl_3_.


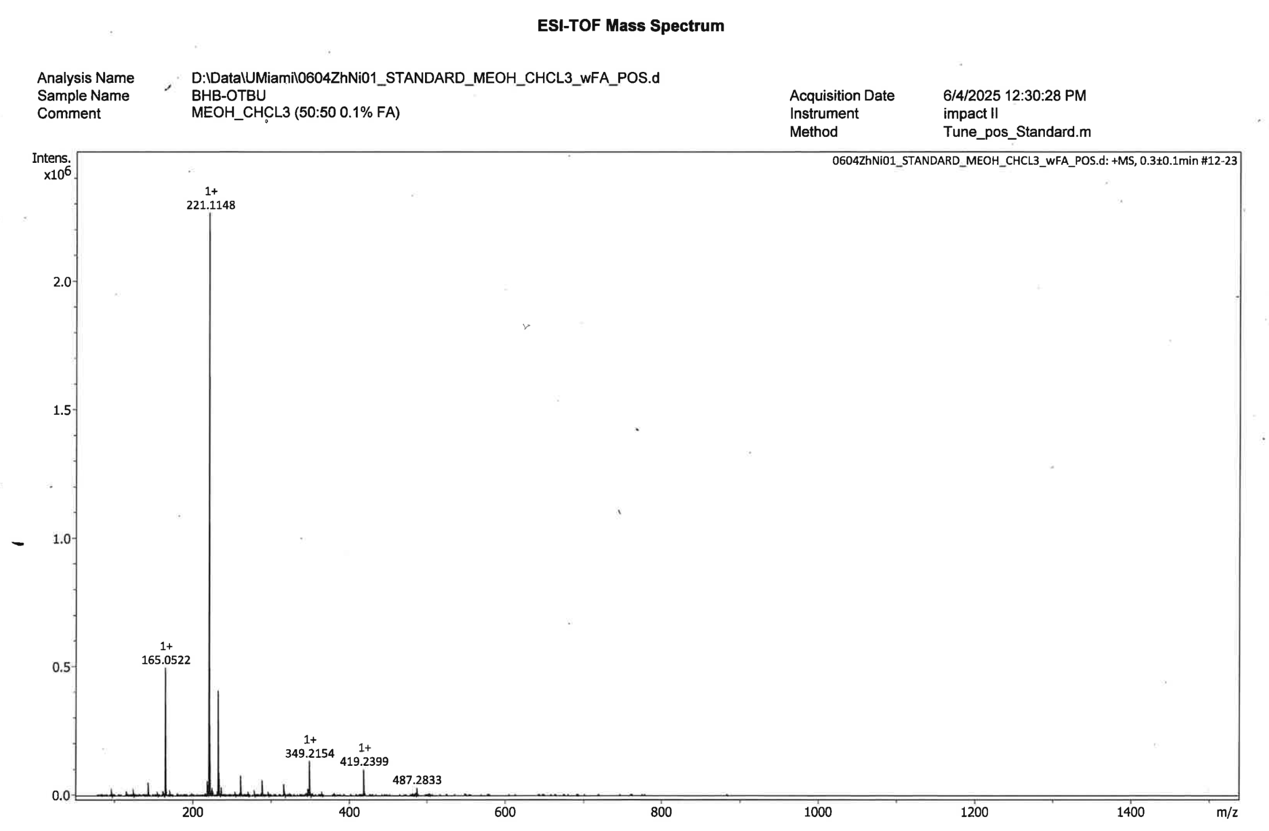


HR-MS spectrum of compound **5**.


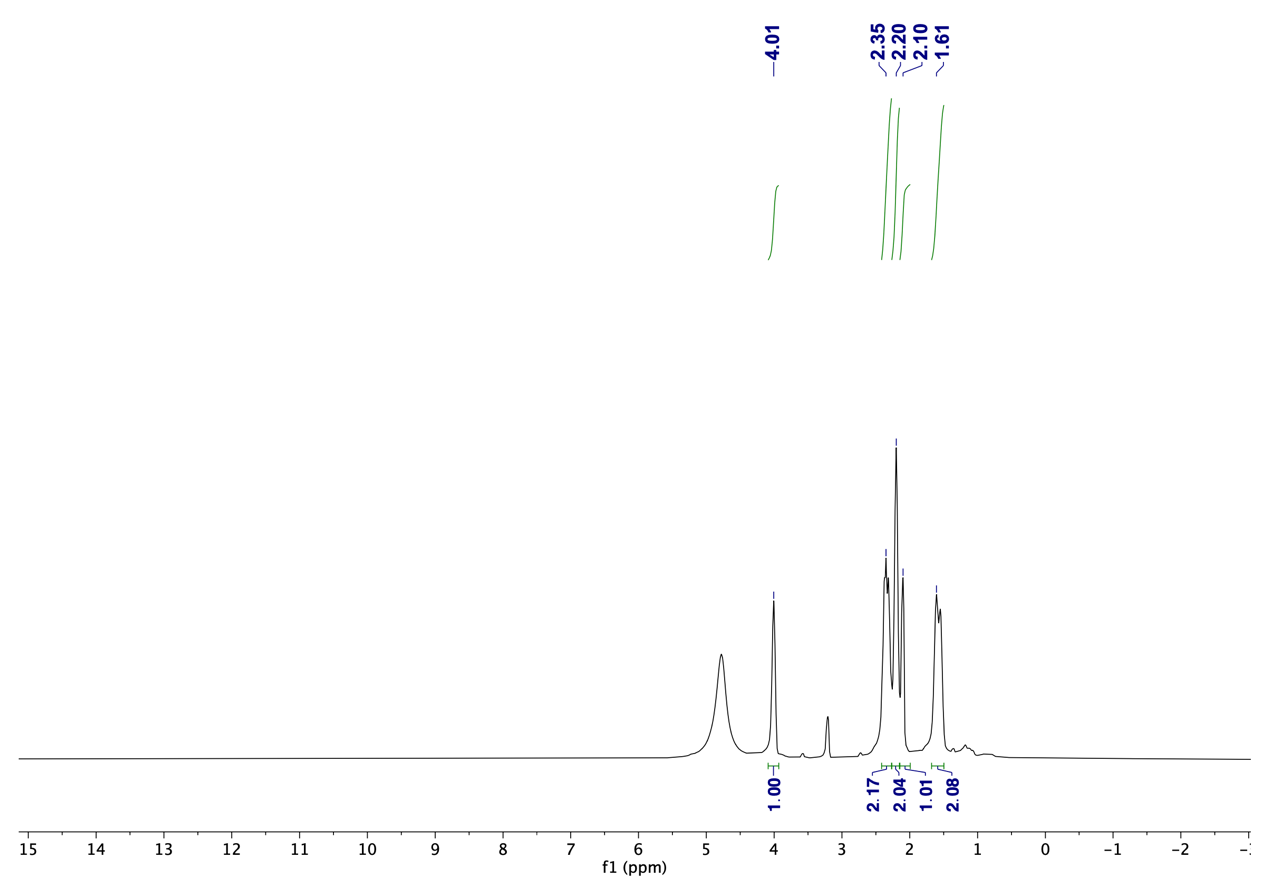


^1^H NMR (500 MHz) spectrum of compound **6** in CD_3_OD.


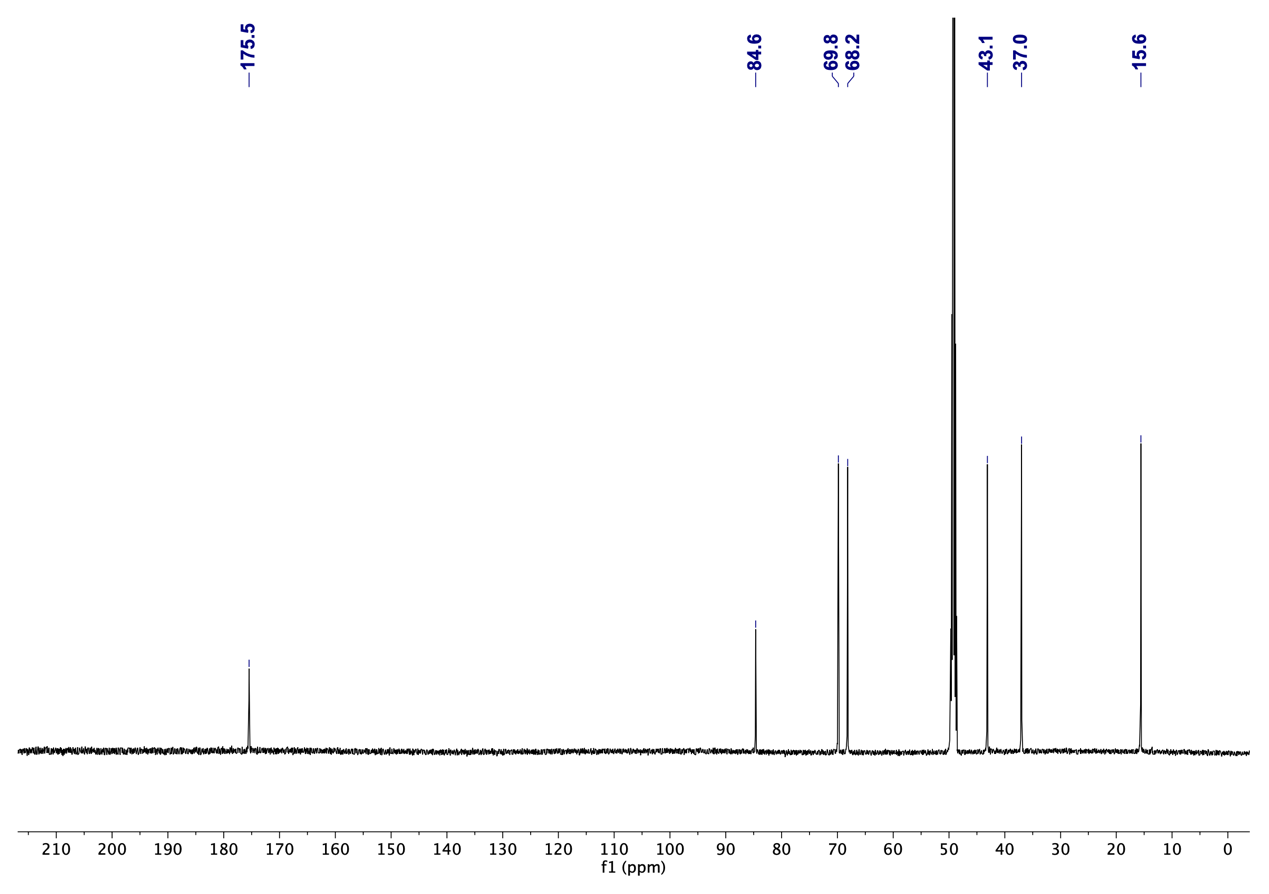


^13^C NMR (126 MHz) spectrum of compound **6** in CD_3_OD.


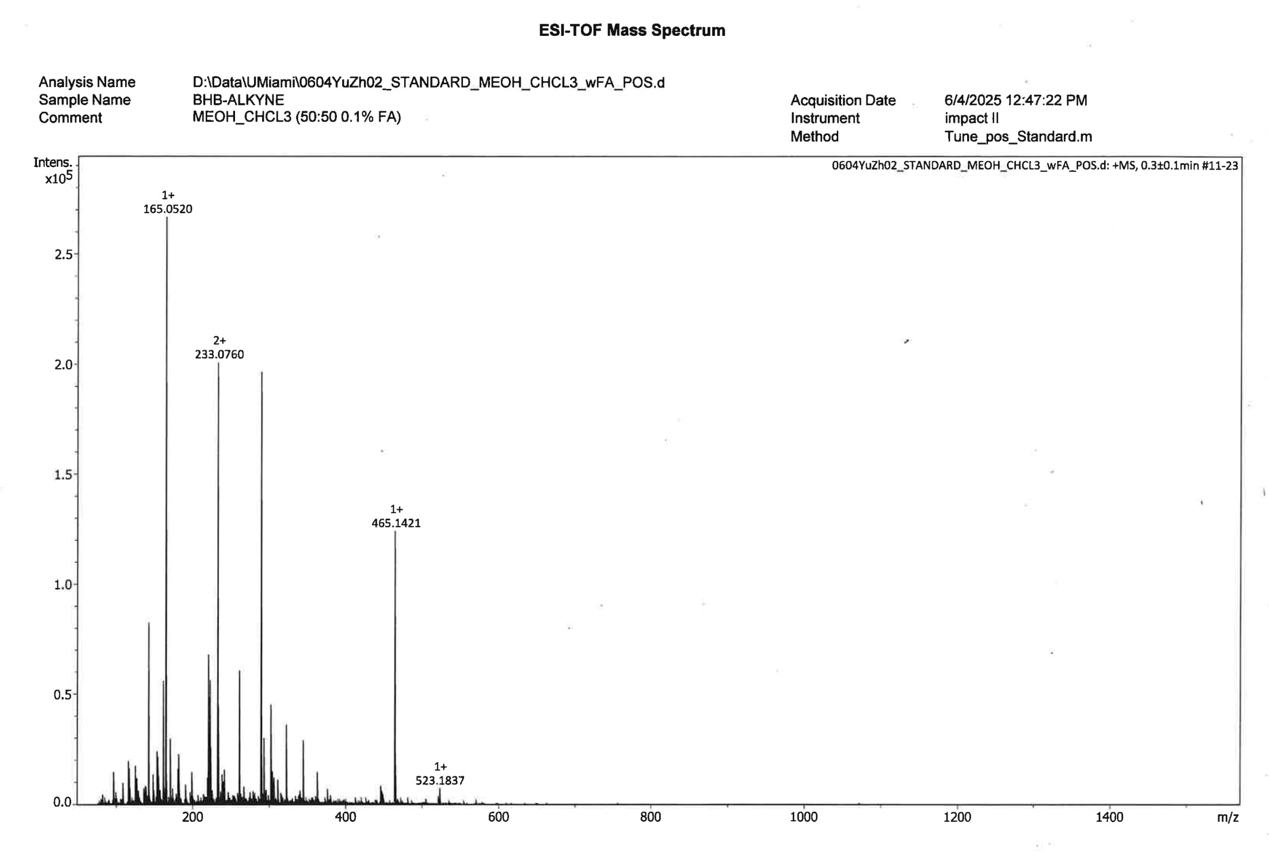


HR-MS spectrum of compound **6**

^1^H NMR (500 MHz) spectrum of compound CcrNFLA in DMSO-*d*_6_.

^13^C NMR (126 MHz) spectrum of compound CcrNFLA in DMSO-*d*_6_.

HSQC spectrum of compound CcrNFLA in DMSO-*d*_6_.


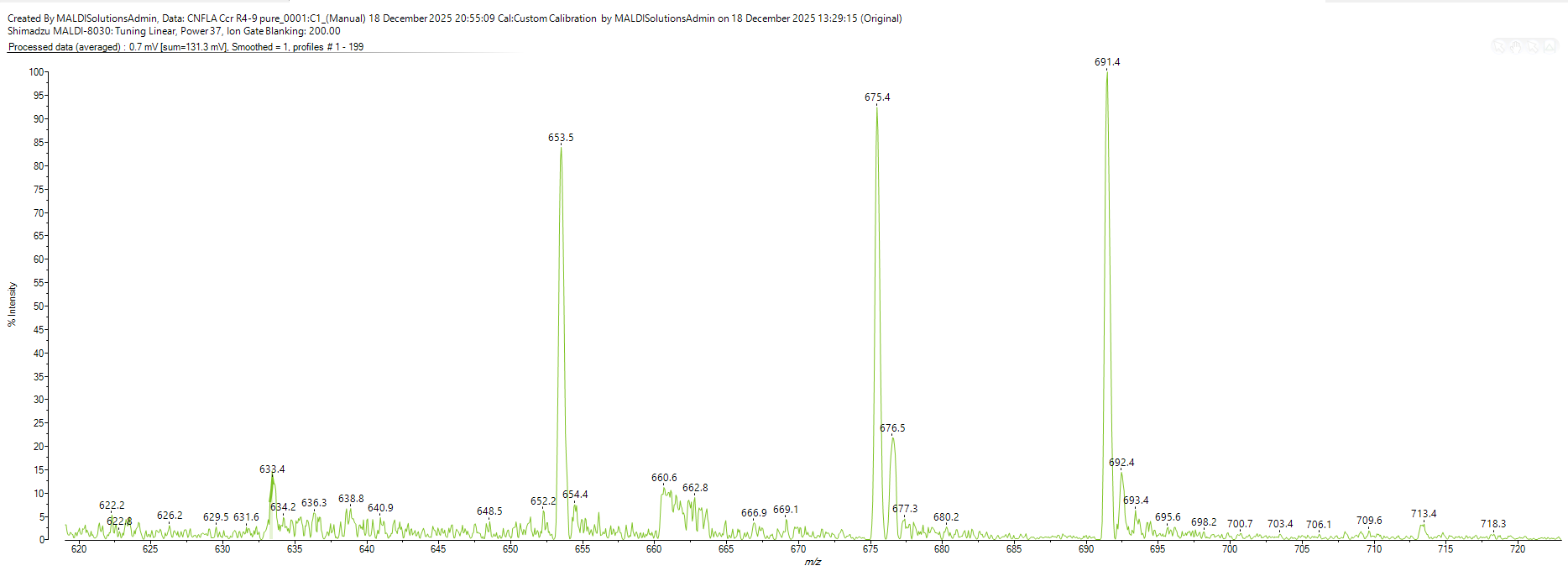


MALDI-TOF MS spectrum of CcrNFLA

1. **References**
2. Kong, A. T.; Leprevost, F. V.; Avtonomov, D. M.; Mellacheruvu, D.; Nesvizhskii, A. I., MSFragger: ultrafast and comprehensive peptide identification in mass spectrometry-based proteomics. Nat. Methods 2017, 14, 513-520.

2. da Veiga Leprevost, F.; Haynes, S. E.; Avtonomov, D. M.; Chang, H. Y.; Shanmugam, A. K.; Mellacheruvu, D.; Kong, A. T.; Nesvizhskii, A. I., Philosopher: a versatile toolkit for shotgun proteomics data analysis. Nat. Methods 2020, 17, 869-870.

3. Yu, F.; Haynes, S. E.; Teo, G. C.; Avtonomov, D. M.; Polasky, D. A.; Nesvizhskii, A. I., Fast Quantitative Analysis of timsTOF PASEF Data with MSFragger and IonQuant. Mol. Cell Proteomics 2020, 19, 1575-1585.

4. Chen, T.; Ma, J.; Liu, Y.; Chen, Z.; Xiao, N.; Lu, Y.; Fu, Y.; Yang, C.; Li, M.; Wu, S.; Wang, X.; Li, D.; He, F.; Hermjakob, H.; Zhu, Y., iProX in 2021: connecting proteomics data sharing with big data. Nucleic Acids Res. 2022, 50, D1522-D1527.

5. Ma, J.; Chen, T.; Wu, S.; Yang, C.; Bai, M.; Shu, K.; Li, K.; Zhang, G.; Jin, Z.; He, F.; Hermjakob, H.; Zhu, Y., iProX: an integrated proteome resource. Nucleic Acids Res. 2019, 47, D1211-D1217.
